# Extensive molecular tinkering in the evolution of the membrane attachment mode of the Rheb GTPase

**DOI:** 10.1038/s41598-018-23575-0

**Published:** 2018-03-27

**Authors:** Kristína Záhonová, Romana Petrželková, Matus Valach, Euki Yazaki, Denis V. Tikhonenkov, Anzhelika Butenko, Jan Janouškovec, Štěpánka Hrdá, Vladimír Klimeš, Gertraud Burger, Yuji Inagaki, Patrick J. Keeling, Vladimír Hampl, Pavel Flegontov, Vyacheslav Yurchenko, Marek Eliáš

**Affiliations:** 1 Department of Biology and Ecology & Institute of Environmental Technologies, Faculty of Science, University of Ostrava, Ostrava, Czech Republic; 2 Department of Biochemistry and Robert-Cedergren Centre for Bioinformatics and Genomics, Université de Montréal, Montreal, Canada; 3 Institute for Biological Sciences, University of Tsukuba, Tsukuba, Japan; 4 Laboratory of Microbiology, Papanin Institute for Biology of Inland Waters, Russian Academy of Sciences, Borok, Russia; 5 Department of Genetics, Evolution and Environment, University College London, London, United Kingdom; 6 Department of Parasitology, Faculty of Science, Charles University, Prague, Czech Republic; 7 Center for Computational Sciences, University of Tsukuba, Tsukuba, Japan; 8 Department of Botany, University of British Columbia, Vancouver, Canada

## Abstract

Rheb is a conserved and widespread Ras-like GTPase involved in cell growth regulation mediated by the (m)TORC1 kinase complex and implicated in tumourigenesis in humans. Rheb function depends on its association with membranes via prenylated C-terminus, a mechanism shared with many other eukaryotic GTPases. Strikingly, our analysis of a phylogenetically rich sample of Rheb sequences revealed that in multiple lineages this canonical and ancestral membrane attachment mode has been variously altered. The modifications include: (1) accretion to the N-terminus of two different phosphatidylinositol 3-phosphate-binding domains, PX in Cryptista (the fusion being the first proposed synapomorphy of this clade), and FYVE in Euglenozoa and the related undescribed flagellate SRT308; (2) acquisition of lipidic modifications of the N-terminal region, namely myristoylation and/or S-palmitoylation in seven different protist lineages; (3) acquisition of S-palmitoylation in the hypervariable C-terminal region of Rheb in apusomonads, convergently to some other Ras family proteins; (4) replacement of the C-terminal prenylation motif with four transmembrane segments in a novel Rheb paralog in the SAR clade; (5) loss of an evident C-terminal membrane attachment mechanism in Tremellomycetes and some Rheb paralogs of Euglenozoa. Rheb evolution is thus surprisingly dynamic and presents a spectacular example of molecular tinkering.

## Introduction

Rheb, a member of the Ras family of GTPases, has attracted significant attention due to its role in a cellular signalling cascade directly relevant to human health. In both metazoans and fungi, Rheb acts as an activator of the protein kinase complex (m)TORC1, a central signalling hub that integrates signals from outside the cell with internal cues to regulate protein synthesis and cell proliferation. Rheb regulates mTORC1 activity in response to various stimuli including growth factors and specific nutrients^1–5^. The mTORC1 signalling is upregulated in a number of human diseases including cancers and is a target for anti-cancer therapy^6^. Mutations disrupting a negative Rheb regulator lead to a tumour syndrome called tuberous sclerosis, and Rheb itself is overexpressed or mutated in various cancers^5,6^.

**Figure 1:**
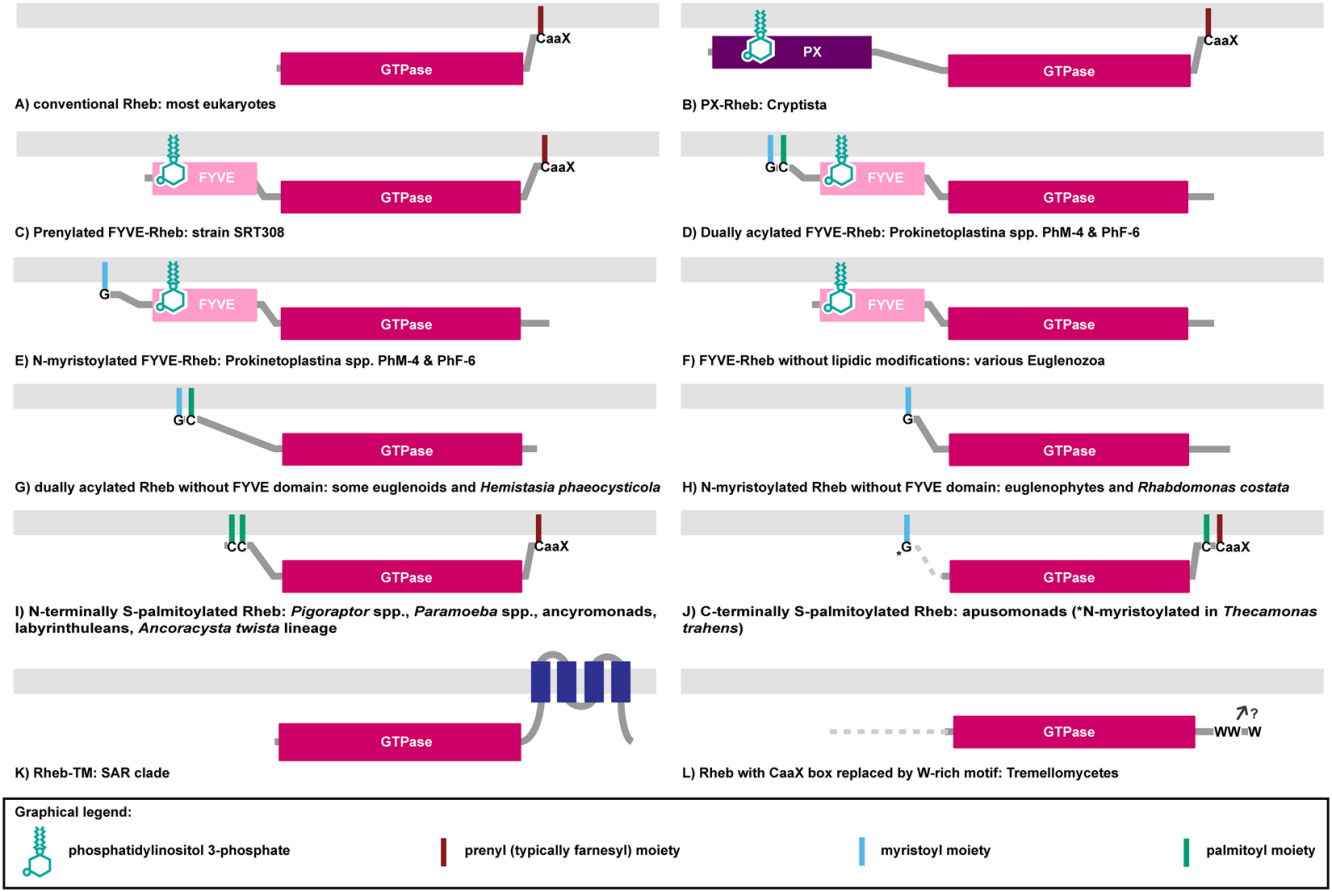
The diversity of membrane-association mechanisms of Rheb proteins. (**A**) Conventional Rheb. (**B**) PX-Rheb in Cryptista (for details see Supplementary Fig. S2). (**C–H**) Unconventional Rheb forms in Euglenozoa and relatives (see Supplementary Fig. S3 for details). (**C**) Prenylated FYVE-Rheb. (**D**) Dually acylated (N-myristoylated and S-palmitoylated) FYVE-Rheb. (**E**) N-myristoylated FYVE-Rheb. (**F**) FYVE-Rheb without lipidic modifications. (**G**) Dually acylated (N-myristoylated and S-palmitoylated) Rheb without the FYVE domain. (**H**) N-myristoylated Rheb without the FYVE domain. (**I**–**J**) S-palmitoylated Rhebs occurring in various protists (for details see Supplementary Fig. S5). (**I**) N-terminally S-palmitoylated Rheb; note that the number of predicted S-palmitoylation sites vary from one to seven in this class (see Supplementary Table S4). (**J**) C-terminally S-palmitoylated Rheb; note that the two proteins known in this category differ in the number of S-palmitoylation sites (one or two; see Supplementary Table S4) and in the presence/absence of N-myristoylation. (**K**) Rheb-TM in the SAR clade (for details see Supplementary Fig. S6). (**L**) Rheb in the Tremellomycetes with the CaaX box replaced by a W-rich motif also possibly (question mark) mediating a membrane association (for details see Supplementary Fig. S7).

The Rheb proteins studied so far conform neatly to the structure typical for Ras-related GTPases in general (Fig. 1A; ref.^5^), which consists of a highly conserved GTPase domain that interacts with specific regulatory and effector proteins, a short hypervariable region, and the C-terminal CaaX box directing attachment of a prenyl moiety (farnesyl in the experimentally studied metazoan and fungal Rhebs) to an invariant cysteine residue^7^. The prenyl moiety anchors Rheb in the membrane (typically lysosomal), and this docking is critical for proper Rheb function, including its ability to stimulate mTORC1^3,5,6^.

Despite the importance of the Rheb protein for eukaryotic cell biology, its evolution has been studied only briefly and in a limited selection of eukaryotes. These analyses established that the Rheb gene was likely present in the last eukaryotic common ancestor (LECA) and that it was secondarily lost in several lineages^8–10^. We set out to evaluate the evolutionary history of the Rheb gene on a much wider scale, taking advantage of recent genome or transcriptome assemblies (including ongoing projects) and focusing on poorly known yet phylogenetically important protist lineages. The bioinformatic analyses were complemented by experimental techniques to test a particular *in silico* insight concerning Rheb in Euglenozoa. The results demonstrate that numerous eukaryotes have reshaped the canonical Rheb structure to change in various ways the membrane attachment mode of the protein. Our work unveils an intriguing example of molecular tinkering^11^ in an evolution of a core eukaryotic protein with implications for our understanding of the eukaryote phylogeny.

## Results and Discussion

We collected and manually curated (verified, completed, or corrected) the sequences of 184 Rheb genes from 154 species representing all major eukaryotic lineages for which large-scale sequence data are available (Supplementary Table S1). Rheb sequences in transcriptome assemblies from several protist were recognized as evident contaminants and excluded from further analyses (Supplementary Table S2). Phylogenetic analysis (Supplementary Fig. S1) confirmed our identification of Rheb orthologs, but showed that the gene previously considered to be a Rheb ortholog in the heterolobosean *Naegleria gruberi*^10^ falls outside the Rheb clade. Furthermore, the sequence does not give Rhebs as its best blastp hits and lacks a highly characteristic arginine residue in the first β-strand of the GTPase domain, which is conserved in nearly all Rheb sequences yet absent from other members of the Ras family (data not shown). Together with its ortholog from *Naegleria fowleri*, it should better be interpreted as a divergent Ras/Rap-related gene. No other apparent Rheb candidate could be identified in either *Naegleria* species spp., whereas other heteroloboseans encode a typical Rheb protein (Supplementary Fig. S1, Supplementary Table S1), suggesting specific Rheb loss in the *Naegleria* lineage. On the other hand, Rheb has been thought to be absent from the kinetoplastid *Bodo saltans*^8^, but it is in fact present (Supplementary Fig. S1, Supplementary Table S1). More than one Rheb gene is found in some species, apparently resulting from lineage-specific duplications (including one in vertebrates leading to the two human Rheb paralogs, RHEB and RHEBL1). The paralogs have typically diverged little from each other, with the interesting exceptions of structurally differentiated Rheb variants in the Euglenozoa and the SAR clade (see below).

**Figure 2:**
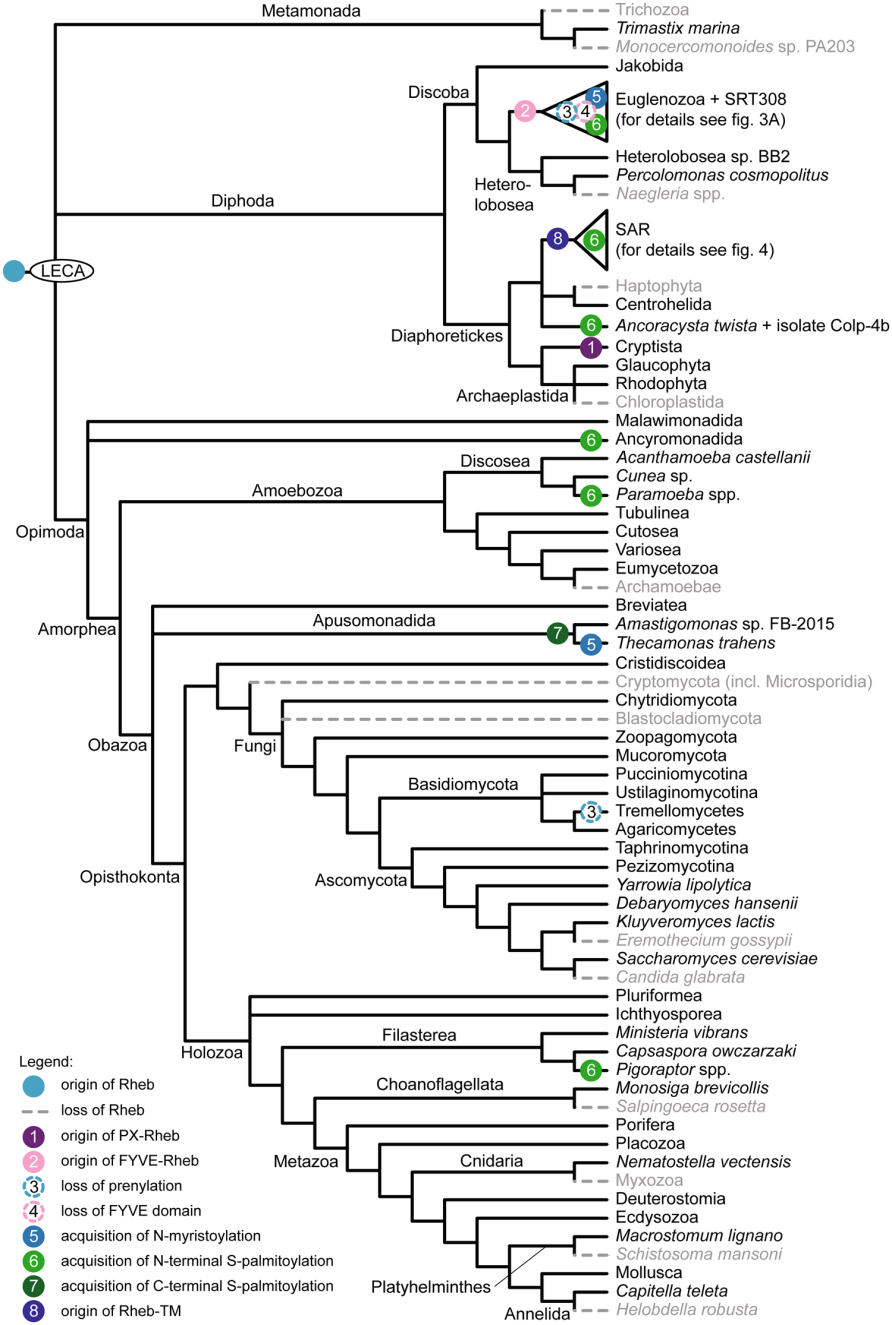
Phylogenetic distribution and main events in the evolution of the Rheb protein. The schematic phylogeny shown is a consensus of recent molecular phylogenies with the last eukaryotic common ancestor (LECA) based on ref.^54^. Note that alternative LECA positions suggested by other authors^55–58^ are all also compatible with Rheb being present already in the LECA. Species representatives for each clade are listed in Supplementary Table S1. A detailed view of the Rheb evolution in Euglenozoa and the SAR clade is provided in Figs 3A and 4, respectively. Only two Rheb-lacking lineages of Saccharomycotina are shown for simplicity; for a more comprehensive view of the Rheb distribution in this group see ref.^8^.

**Figure 3:**
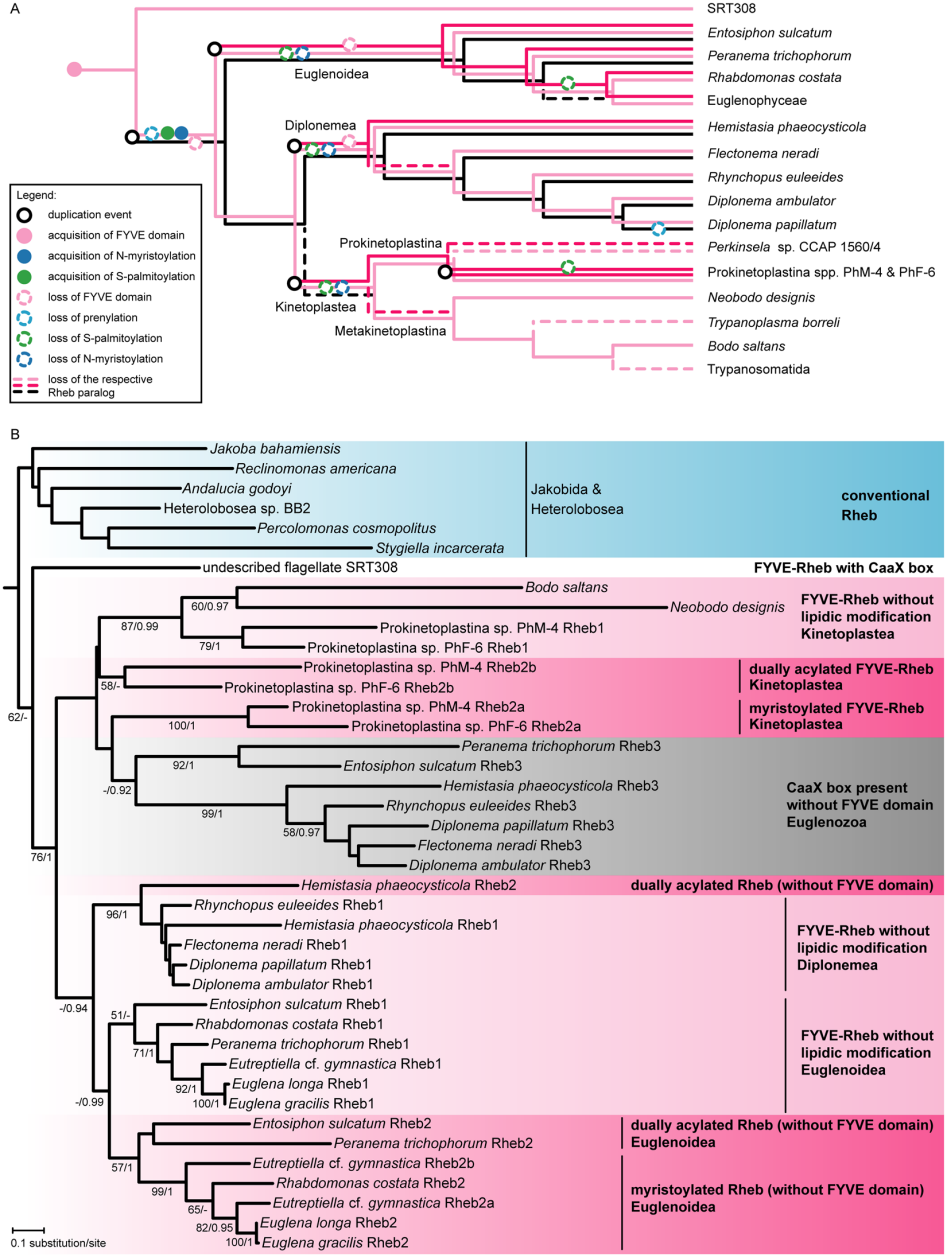
Rheb evolution in the Euglenozoa. (**A**) Inferred origin, loss, and modifications of Rheb paralogs in the Euglenozoa. Relationships of the taxa are based on ref.^59^ and ref.^60^; note that *Flectonema neradi*^61^ was previously referred to as *Diplonema* sp. 2. The placement of the two unnamed Prokinetoplastina species is based on an unpublished phylogenomic analysis (Tikhonenkov *et al*. in preparation). The Euglenophyceae comprise *Euglena* spp. and *Eutreptiella* cf. *gymnastica*. (**B**) Maximum likelihood phylogenetic tree (RAxML-HPC2, LG4X + Γ substitution model) of Rheb proteins in the Euglenozoa and relatives. Support values at branches correspond bootstrap values (>50%) from the ML analysis and to posterior probabilities (>0.9) from an analysis by MrBayes. Sequence identifiers are provided in Supplementary Table S1.

**Figure 4:**
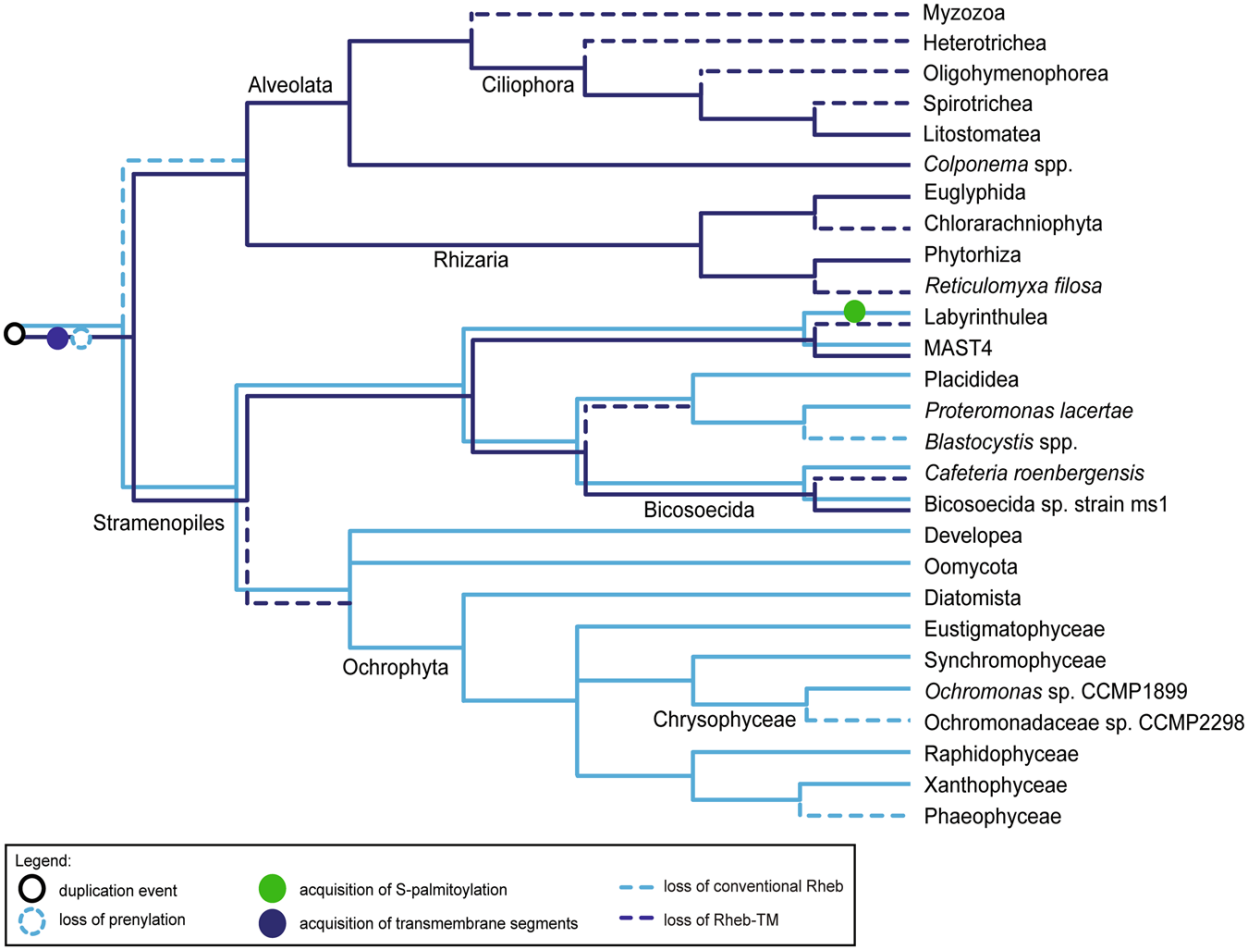
A schematic of Rheb evolution in the SAR clade. The figure shows the most parsimonious interpretation of the taxonomic distribution, sequence features, and phylogenetic relationship of Rheb proteins in the group. The phylogenetic relationships of the taxa are depicted based on the most recent molecular phylogenetic and phylogenomic analyses. Relationships of the taxa are based on refs^33,62,63^. Species representing the broader clades indicated in the tree are listed in Supplementary Table S1.

Our expanded sampling reinforces the notion that Rheb was already present in the LECA (Fig. 2) but also documents further the prevalence of secondary losses of this GTPase (inferred from high-quality genome assemblies or transcriptomic data for multiple representatives of the given taxon). For example, within the Metazoa, Rheb is missing not only from some platyhelminths, as previously noticed^8^, but also from the leech *Helobdella robusta* and myxozoans (divergent parasitic cnidarians). Other newly recognized Rheb-lacking taxa include the model choanoflagellate *Salpingoeca rosetta*, haptophytes, and brown algae (Phaeophyceae). The most parsimonious interpretation of Rheb distribution therefore implies well over 30 independent gene losses (these include differential losses of Rheb paralogs in the Euglenozoa and SAR clade; Figs 2–4). The loss events map to ancestors of large eukaryotic clades (e.g., Chloroplastida or Haptophyta), as well as to terminal branches (e.g., at least five independent Rheb losses among Saccharomycotina yeasts; see ref.^8^). Recurrent loss is not uncommon in the larger superfamily of Ras GTPases^12^ but, except for isolated cases (e.g., ref.^13^), the functional or evolutionary significance of losses is not understood and does not apparently correlate with organismal life style or cellular organization.

In contrast to the elusive patterns of Rheb distribution, analyses of the Rheb protein architectures unveiled surprising diversity of evolutionary changes with readily interpretable important functional implications. These comprise secondary modifications of the regions flanking the central GTPase domain that affect the mechanism of the protein-membrane interaction. Below we discuss the structure, distribution and evolutionary origin of each of the novel Rheb forms identified.

Let us first discuss the Rheb structure in the recently delineated clade Cryptista uniting cryptomonads, katablepharids, and *Palpitomonas bilix*^14,15^. We found that Rhebs in all cryptist lineages share a unique structure in which a PX domain has been accreted to N-terminus of the GTPase domain (Figs 1B and 2, Supplementary Fig. S2, Supplementary Table S1). This represents the first candidate synapomorphy for the Cryptista, a grouping so far supported solely by phylogenomic trees. The PX domain specifically binds phosphatidylinositol 3-phosphate (PI3P; ref.^16^), a phospholipid localized to compartments of the endocytic pathway in diverse eukaryotes^17–19^. The presence of the PX domain thus indirectly suggests that cryptist Rheb is localized to the endocytic pathway (similar to Rheb localization in metazoan cells).

Unexpectedly, we unveiled a second, independent case of a Rheb fusion involving an N-terminal PI3P-binding module – the FYVE domain functionally similar yet completely unrelated to PX^20^. This FYVE-Rheb (Fig. 1C–F) was encountered in all three main groups of Euglenozoa, i.e., euglenoids, diplonemids, and free-living kinetoplastids (the endosymbiotic *Perkinsela* and parasitic trypanosomatids lack Rheb; Fig. 3A, Supplementary Table S1, Supplementary Fig. S3). In addition, we documented FYVE-Rheb in the recently discovered and hitherto undescribed flagellate SRT308 that constitutes a new sister lineage to the Euglenozoa^21^. Since more distantly related lineages (jakobids and heteroloboseans) possess conventional Rhebs (Supplementary Table S1), the fusion of the FYVE domain to Rheb is an apparent synapomorphy of the Euglenozoa and SRT308 (Figs 2 and 3A). Notably, the FYVE-Rheb protein from SRT308 has kept the characteristic C-terminal prenylated CaaX motif (Fig. 1C), suggesting that it associates with the membrane via both termini (like the cryptist PX-Rheb). In contrast, all euglenozoan FYVE-Rhebs lack the C-terminal prenylation motif (Fig. 1D–F, Supplementary Fig. S3).

Rheb evolution in the Euglenozoa is nevertheless more complex. Our transcriptomic data revealed that two so far unidentified species representing the basal kinetoplastid lineage Prokinetoplastina each possess three FYVE-Rheb paralogs, two of which carry a predicted N-terminal myristoylation signal, whereas euglenoids and the diplonemid *Hemistasia phaeocysticola* exhibit putatively myristoylated paralogs lacking the FYVE domain (Supplementary Fig. S3; Supplementary Table S3). N-terminal glycine myristoylation (N-myristoylation) is a common eukaryotic protein modification and it is known to mediate membrane attachment in Ras superfamily proteins, particularly Arf and Arl^22^. However, to our knowledge, it has not yet been reported in GTPases belonging to the Ras family itself. To test whether a Rheb can be myristoylated *in vivo*, the putatively myristoylated Rheb gene from *Euglena longa* was co-expressed in *Escherichia coli* with an *E*. *longa* gene encoding the N-myristoyl transferase (NMT), an enzyme responsible for protein N-terminal myristoylation^23^. Mass spectrometry detected a clear signal for the presence of myristoylation when Rheb was co-expressed with NMT, but not in the control without NMT (Supplementary Fig. S4). The positive experimental result gives credence to our *in silico* prediction of N-myristoylation in other euglenozoan Rhebs, most of which in fact score better as putative N-myristoylation targets than the *E*. *longa* protein (see Supplementary Table S3).

N-myristoylation of proteins is frequently accompanied by S-palmitoylation of cysteine residues in the vicinity of the myristoylated glycine to provide an additional lipidic anchor facilitating their membrane association^24^, as also documented in some Ras superfamily GTPases^25,26^. Indeed, the N-terminal extension of a subset of N-myristoylated euglenozoan Rhebs contains cysteine residues that are putatively palmitoylated (Supplementary Fig. S3, Supplementary Table S4). Altogether, we found five different configurations of the N-terminal membrane-attachment devices in euglenozoan Rhebs: dual acylation plus the FYVE domain (one paralog from Prokinetoplastina spp.; Fig. 1D), N-myristoylation only plus FYVE (another paralog from Prokinetoplastina spp.; Fig. 1E), FYVE only (some paralogs in all three main euglenozoan lineages; Fig. 1F), dual acylation without FYVE (one of the paralogs in basal euglenoids and *Hemistasia*; Fig. 1G), and N-myristoylation only (one of the paralogs in euglenophytes and *Rhabdomonas costata*; Fig. 1H).

To make matters even more complex, diplonemids and basal euglenoids encode an additional Rheb paralog with a poorly conserved N-terminus (in terms of both the length and sequence) lacking any apparent membrane-association devices, yet having a conserved C-terminal CaaX box (Supplementary Fig. S3). Curiously, the *Diplonema papillatum* Rheb protein of this group has lost the prenylation motif (this does not seem to be a sequencing or prediction artefact, see Supplementary Table S1). This raises the question of whether and how it associates with the membrane.

The length of the Rheb proteins limits the resolution of their phylogeny, but a phylogenetic analysis of euglenozoan sequences (Fig. 3B) does provide several important clues for the interpretation of their evolutionary history (illustrated in Fig. 3A). All euglenozoan paralogs are monophyletic and sister to the prenylated FYVE-Rheb in the SRT308 isolate. This indicates duplication of the ancestrally single, prenylated FYVE-Rheb in the euglenozoan stem lineage. One of the two paralogs subsequently dispensed with the FYVE domain and was later lost in kinetoplastids and some euglenoids. The other paralog lost the CaaX box and hence prenylation, which was accompanied by the acquisition of a dual N-terminal acylation. According to the phylogenetic analysis, the dually acylated FYVE-Rheb was then independently duplicated in different euglenozoan. In euglenoids, one of the two copies lost the acylation whereas the other lost the FYVE domain and later (in a common ancestor of euglenophytes and *R*. *costa*) even the S-palmitoylation, leaving the N-myristoylation as the protein’s sole means of membrane attachment. A similar scenario was paralleled in diplonemids, resulting in a FYVE-Rheb copy lacking acylation and a dually acylated paralog without the FYVE domain, the latter retained only in *Hemistasia*. In kinetoplastids the Rheb phylogeny implies a duplication preceding the separation of the Prokinetoplastina and the Metakinetoplastina. One copy, kept by some members of both lineages, dispensed with the N-terminal acylation, whereas the second copy, preserving the ancestral organization, was apparently lost in the Metakinetoplastina yet duplicated in the Prokinetoplastina lineage, with one of the duplicates losing S-palmitoylation. The deduced evolutionary history of euglenozoan Rhebs, unparalleled in complexity by any other eukaryotic group, may suggest specific functional circumstances driving the Rheb evolution in this taxon. Unfortunately, the Rheb absence in trypanosomatids precludes using well-established model organisms such as *Trypanosoma brucei* for functional studies on the euglenozoan Rheb.

Remarkably, we found five additional protist groups that seem to have independently acquired S-palmitoylation in their Rheb N-terminal region. Specifically, Rhebs in the genera *Pigoraptor* (Filasterea) and *Paramoeba* (Amoebozoa), ancyromonads, labyrinthuleans, and members of the recently described novel eukaryotic lineage typified by *Ancoracysta twista* (see ref.^27^) all keep the C-terminal CaaX box but at the same time exhibit an N-terminal extension with one or (typically) more cysteine residues predicted as S-palmitoylation targets (Figs 2 and 4, Supplementary Table S4). This prediction is supported by the fact that the cysteine residues are located at similar positions in the sequences of the five taxa (Supplementary Fig. S5). N-myristoylation is likely absent: most sequences lack a suitable motif and the glycine residue at the second position of the *A*. *twista* Rheb is predicted as an unlikely target (Supplementary Table S3, Supplementary Fig. S5). N-terminal S-palmitoylation without myristoylation is not unprecedented among Ras superfamily GTPases (e.g., ref.^25,28^), but the predicted co-occurrence of N-terminal S-palmitoylation and C-terminal prenylation in Rhebs of the five protist groups (Fig. 1I) is novel.

On the other hand, S-palmitoylation is a well-established modification of the C-terminal hypervariable region of some Ras and Rap proteins, specifically of one or two cysteine residues upstream of the prenylation motif^29^. Opisthokont Rhebs were noted to lack this modification^3,5^, and this is apparently typical of eukaryotes, since candidate palmitoylatable cysteines are missing from the C-terminal region of the vast majority of Rheb sequences in our sample. However, Rheb proteins from apusomonads (represented by two distantly related species) do exhibit one (*Amastigomonas* sp.) or two (*Thecamonas trahens*) cysteine residues at positions equivalent to S-palmitoylated sites in Ras or Rap proteins (Supplementary Fig. S5), and these positions are predicted to be S-palmitoylated (Supplementary Table S4). This suggests that the apusomonad Rheb may have adopted a dual C-terminal lipidic modification convergently to some other members of the Ras family (Fig. 1J). Strikingly, the Rheb protein from *T*. *trahens* is also predicted (by three independent tools) to carry an N-terminal myristoylation motif (Supplementary Fig. S5, Supplementary Table S3), suggesting that it receives three different lipidic modifications.

SAR is a huge, robustly supported eukaryotic clade comprised of three principal lineages: stramenopiles, alveolates, and rhizarians^14,30,31^. Organisms belonging to the SAR clade are extremely diverse and lack a defined morphological synapomorphy. It was thus interesting to find out that members of all three SAR lineages share a novel Rheb form with a unique structure of the C-terminal region. Specifically, the C-terminus of this form (Rheb-TM) is extended by a region that includes four predicted transmembrane segments, which would provide a stable membrane anchor (Fig. 1K, Supplementary Fig. S6). Interestingly, the cysteine residue of the CaaX box, presumably present in the Rheb-TM ancestor before accretion of the C-terminal extension, is conserved in most Rheb-TM sequences (Supplementary Fig. S6). Why this residue has remained conserved is unclear, since its internal position within the Rheb-TM protein renders it an unlikely substrate for prenylation given the specificity of prenyltransferases for C-terminally located cysteine residues^32^.

Among stramenopiles we found Rheb-TM in a bicosoecid and a member of the uncultured clade MAST4, but both organisms possess a second, conventional Rheb paralog found also in other stramenopiles. All alveolates and rhizarians seem to have lost the conventional Rheb, but sequences of Rheb-TM were found in transcriptome assemblies from colponemids, recently recognized as basal alveolates^33^, ciliates of the class Litostomatea, and five rhizarians (Supplementary Table S1). No Rheb-TM was found outside the SAR clade: members of its putative sister group, the Haptista^14^, possess only the conventional Rheb (centrohelids; based on transcriptomic data) or lack Rheb altogether (haptophytes; Supplementary Table S1). The restriction of Rheb-TM to the SAR clade, its co-occurrence with the conventional Rheb in some stramenopiles, and the presence of Rheb-TM as the only Rheb variant in alveolates and rhizarians point to a Rheb duplication in the SAR stem lineage, followed by a modification of the C-terminal region in one of the paralog and subsequent differential loss (Fig. 4). We thus propose that the Rheb-TM paralog is a second identified synapomorphy of the SAR clade (the first being a novel Rab1 paralog; ref.^34^).

Finally, Rheb proteins in Tremellomycetes (a group of fungi including the human parasite *Cryptococcus neoformans*) have replaced the C-terminal CaaX box with a motif including two or three tryptophan residues (Fig. 1L, Supplementary Fig. S7). This indicates loss of prenylation and the existence of an alternative mode of membrane attachment in Rheb proteins in this group. Only some tremellomycete Rhebs have an N-terminal extensions preceding the GTPase domain, but in just a minor subset of those the extension includes cysteine residues that could be S-palmitoylation targets (data not shown). We thus speculate that these Rhebs are recruited to the membrane via a novel mechanism relying on the C-terminal tryptophan motif.

## Conclusions

Our analysis unveiled a curious example of an ancestral and widely distributed eukaryotic gene with a central cellular function that exhibits a surprising level of evolutionary plasticity. The various modifications of the Rheb protein at the N- or C-terminal side of the GTPase domain affecting the way of its interaction with the membrane provide a prime example of molecular tinkering in protein evolution. Analyses of other Ras superfamily GTPases reported so far, admittedly less exhaustive in terms of taxon sampling, did provide examples of novel domain architectures or changes in post-translation modifications. Such examples include a Metazoa-specific subgroup of the Rabs that has acquired a very long N-terminal extension including several functional domains^35^ or a Rab5-related paralog in Chloroplastida and some alveolates that has dispensed with the C-terminal geranylgeranylation canonical for Rab family members and instead is dually acylated at the N-terminus^26^. However, such occasional modifications typically affect “neofunctionalized” lineage-specific paralogs newly arisen by duplication of a particular ancestral GTPase. In contrast, Rheb modifications are not necessarily linked to gene duplication, and if they are, the duplicated modified form can replace the original one (as is the case of Rheb-TM in Alveolata and Rhizaria). To the best of our knowledge, there is no other eukaryotic GTPase orthogroup that would exhibit nearly as extensive structural variation across the eukaryote phylogeny as Rheb. This makes the evolutionary fate of the Rheb different from other broadly conserved ancestral eukaryotic GTPases.

Unfortunately, sequence analyses alone cannot answer the fundamental question of why ultimately did Rhebs in different lineages accrete PI3P-binding domains, lost the original prenylation, gained different new lipidic modifications, or switched from peripheral to transmembrane proteins. Direct biochemical and cell biological approaches are needed to illuminate the functional differences between the conventional Rheb studied in common model organisms and the non-conventional forms described here, although we note that only a few of the organisms with non-canonical Rheb structures are presently amenable for functional analyses.

Our study also demonstrates the power of dense taxonomic sampling: most of the interesting departures from the conventional Rheb structure occur in little studied or only recently discovered lineages. Only thanks to data from such organisms we could capture important “missing evolutionary links”, namely the prenylated FYVE-Rheb in the flagellate SRT308 and the acylated FYVE-Rhebs in the unidentified Prokinetoplastina species that proved critical for inferring the course of Rheb evolution in Euglenozoa. Quickly improving sampling of eukaryotic genomes or transcriptomes will enable us to further refine our understanding of the origin and distribution of particular Rheb forms and possibly to discover new, so far unimaginable variants.

## Methods

### Assembling a taxonomically-wide curated dataset of Rheb sequences

Rheb genes/proteins were identified by BLAST searches^36^ against a number of databases, including our unpublished transcriptome or genome sequence data from several protist species (for sources of the sequences analysed in this study see Supplementary Table S1). The human RHEB protein (RefSeq accession number NP_005605.1) was used as the primary query for the searches, other queries were used depending on the specific purpose of the search (e.g., using a Rheb sequence from a closely related taxon when double-checking the apparent Rheb presence in a particular organism). Databases of predicted protein sequences from the respective species, if available, were searched using blastp. When only unannotated genome or transcriptome assemblies were available and in cases where no candidate Rheb ortholog was found in the protein sequence database, tblastn was used to search the respective nucleotide sequence databases. Candidate Rheb sequences were evaluated by blastp or blastx searches against a large in-house database of manually curated Ras superfamily sequences. Sequences more similar to known Rhebs than to other Ras superfamily members and (very few) sequences for which the blast-based assignment was not clear-cut were further examined by phylogenetic analyses including also selected reference sequences representing other Ras family members (Supplementary Fig. S1). The list of the bona fide Rheb sequences identified and analysed in this study is provided in Supplementary Table S1. We cannot exclude the possibility that some extremely divergent Rheb orthologs were not identified by our approach. Some sequences in transcriptome assemblies from several species were discarded as obvious contaminants (Supplementary Table S2). Their likely sources were revealed by considering sequence similarity to other Rheb sequences and/or by checking the identity of 18S rRNA sequences in the respective assemblies.

Existing protein sequence predictions were evaluated and, when necessary, corrections were introduced guided by the respective transcript sequences (expressed sequence tags – ESTs, or transcript shotgun assemblies – TSA) and/or by sequence conservation among orthologous Rheb proteins. Transcript contigs or unannotated genome sequences were conceptually translated in all six reading frames and the coding sequence was deduced manually. In several cases the existing nucleotide sequences that proved truncated at the 5′- or 3′-end were extended by iterative blastn searches of the raw sequencing reads from the respective sequencing project in the Short Read Archive database at NCBI (https://www.ncbi.nlm.nih.gov/sra) or in our own sequence database to assemble a complete coding sequence of the genes. All the corrections are specified for the respective genes in Supplementary Table S1.

The 3′-end of the Rheb cDNA from *Hemiarma marina* was completed by 3′-RACE using the GeneRacer Kit (Thermo Fisher Scientific) was used, following manufacturer’s instructions. Two primers, Hm1 (5′-GGTACATCACAGGTCCCTCGAGTGC-3′) and Hm2 (5′-GCAAGTGGGAAGTGACGAAGGATAC-3′), were designed on the basis of the nucleotide sequence of *H*. *marina* Rheb transcript contig. The SuperScript III reverse transcriptase was used to synthesize the first-strand cDNA, initiated by the GeneRacer Oligo dT Primer (5′-GCTGTCAACGATACGCTACGTAACGGCATGACAGTG(T)24–3′) supplied in the kit. The cDNA sample was then subjected to the first PCR using Hm1 and the GeneRacer 3′ Primer (5′-GCTGTCAACGATACGCTACGTAACG-3′). The amplicons from the first PCR then served as the template DNA in a nested (second) PCR with Hm2 and the GeneRacer 3′ Nested Primer (5′-CGCTACGTAACGGCATGACAGTG-3′). The amplified DNA fragments from the second PCR were cloned into the pGEMT-Easy vector (Promega) and subjected to Sanger sequencing. All the newly reported Rheb sequences were deposited at GenBank with accession numbers MG702351-MG702364 and MG905949-MG905973.

### Bioinformatic and phylogenetic analyses of Rheb sequences

Structural and functional elements of Rheb proteins were predicted using a variety of bioinformatics tools. Conserved protein domains were identified using searches of Pfam (http://pfam.xfam.org/; ref.^37^) and the Conserved Domains database (https://www.ncbi.nlm.nih.gov/Structure/cdd/wrpsb.cgi; ref.^38^). Prediction of putative transmembrane segments was done primarily using the TMHMM Server v. 2.0 (http://www.cbs.dtu.dk/services/TMHMM/), but the analysis was complemented by TMpred (https://www.ch.embnet.org/software/TMPRED_form.html) that could recognize the fourth expected transmembrane segment in the *Leptophryx vorax* Rheb protein not predicted by TMHMM (Supplementary Fig. S6).

N-terminal myristoylation (N-myristoylation) sites were predicted using the ExPASy Myristoylator tool (http://web.expasy.org/myristoylator/; ref.^39^), NMT - The MYR Predictor (http://mendel.imp.ac.at/myristate/SUPLpredictor.htm; ref.^40^), and GPS-Lipid (http://lipid.biocuckoo.org/webserver.php; ref.^41^). Only glycine residues at the second position of the full-length protein sequence (immediately downstream of the initial methionine) were considered as candidate myristoylated sites, since internal glycines can be myristoylated only if they are exposed at the N-terminus by protein cleavage (for which no evidence is available in case of any Rheb protein). Only N-myristoylation sites recognized by at least two of the tools were considered as significant candidates (see Supplementary Table S3). S-palmitoylation was predicted using SeqPalm (http://lishuyan.lzu.edu.cn/seqpalm/; ref.^42^), the CKSAAP-Palm programme (http://doc.aporc.org/wiki/CKSAAP-Palm; ref.^43^), PalmPred (http://proteininformatics.org/mkumar/palmpred/index.html; ref.^44^), GPS-Lipid, and WAP-Palm (http://bioinfo.ncu.edu.cn/WAP-Palm.aspx; ref.^45^). Cysteine residue within conserved functional domains (GTPase, FYVE) and those in the C-terminal CaaX boxes (which are almost certainly prenylated) were a priori excluded as S-palmitoylation sites. To reduce the rate of false positive prediction of S-palmitoylation sites only those predicted by at least three tools were considered as significant candidates (Supplementary Table S4). Note that WAP-Palm cannot predict S-palmitoylation for cysteine residues too close to protein termini, as it requires the evaluated site to be flanked by at least nine amino acid residues on both sides. All acylation predictors were used with the default setting, except for GPS-Lipid that had the “Threshold” set to “High” (instead of the default “Medium”).

Conserved features of different Rheb subgroups were also evaluated by visual inspection of multiple sequence alignments built using MAFFT version 7 (http://mafft.cbrc.jp/alignment/server/; ref.^46^). For presentation purposes alignments were processed using the programme CHROMA (http://www.llew.org.uk/chroma/; ref.^47^). Phylogenetic analyses were carried out using multiple sequence alignments built with MAFFT and trimmed manually (with the aid of GeneDoc v2.7; http://genedoc.software.informer.com/2.7/) to remove poorly conserved regions (protein alignments used for phylogenetic analyses are available upon request). Maximum likelihood trees were inferred using RAxML-HPC2 (with the LG4X + Γ substitution model and rapid bootstrapping). Bayesian trees were inferred using MrBayes v3.2.6^48^ with the WAG + Γ + I substitution model, 1 × 10^6^ generations, sampling frequency of 100, and burn-in fraction of 25%. The phylogenetic analyses were run at the CIPRES Portal (http://www.phylo.org/sub_sections/; ref.^49^). The trees were visualized using iTOL (http://itol.embl.de/; ref.^50^) and further adjusted with a graphical editor.

### Preparation of DNA constructs for heterologous expression of genes from Euglena longa

*Euglena longa* strain CCAP 1204–17a was cultivated statically under constant illumination at 23 °C in Cramer-Myers medium^51^ supplemented with ethanol (0.8% v/v). The cultures of *E*. *longa* were not completely axenic, but the contaminating bacteria were kept at as low a level as possible. RNA was isolated using the RNeasy Plus Universal Mini Kit (Qiagen, Hilden, Germany). cDNA synthesis was carried out with random hexanucleotide primers using the Transcriptor First Strand cDNA Synthesis Kit (Roche, Basel, Switzerland). The complete coding sequences (CDSs) of the genes EloRheb2 (putatively myristoylated Rheb paralog, Supplementary Table S1) and EloNmt (N-myristoyl transferase; the sequence deposited at GenBank with the accession number MG905974) were amplified using the following primers: EloRheb2 – Rheb_fw_NdeI (5′-GTAGGACATATGGGCAATTCAAGTGACAAGGAGAA-3′) and Rheb_rev_SpeI (5′-GCAATACTAGTGCTGCTCTGGAACATCCCCAT-3′); EloNmt – NMT_fw_NcoI (5′-GAAAACCATGGGGTATCCATATGATGTTCCAGATTATGCTGAACCGGCCCTGCCCGGGCGC-3′; introducing the HA-tag at the N-terminus of the protein) and NMT_rev_XhoI (5′-GAAACCTCGAGCTACAACAAAACCAGGGCCATGTC-3′). The Expand High Fidelity PCR System (Roche) was used with the following PCR conditions: 94 °C for 2 min; 30 cycles of 94 °C for 15 sec, 65 °C for 30 sec, 72 °C for 1 min, and the final extension at 72 °C for 7 min. The amplified CDSs were cloned into the vectors pET-42b+ conferring Kan resistance or pET-14b conferring Amp resistance (both from Merck Millipore, Darmstadt, Germany), depending on the gene. The amplified EloRheb2 CDS was cloned into the pET-42b+ vector between *Nde*I and *Spe*I restriction sites in frame with the downstream His- and S-tag. The amplified EloNmt CDS was cloned into the MCS of the pET-14b vector between *Nco*I and *Xho*I restriction sites. All enzymes necessary for cloning were from New Englad Biolabs (Ipswitch, USA). All constructs were verified by sequencing (Macrogen Europe, Amsterdam, Netherlands).

### Protein expression, purification and mass spectrometry

BL21 (DE3) pLysS *Escherichia coli* were transformed and used for protein production in standard conditions. For production of myristoylated EloRheb2 a previously published protocol was followed^52^. Briefly, myristic acid (Sigma-Aldrich, St. Louis, USA) as the NMT substrate was added 10 min before induction to a final concentration of 50 μM. Myristic acid was freshly prepared as a 5 mM stock solution (pH = 9) containing 0.6 mM BSA (Sigma-Aldrich). The same treatment, yet without adding myristic acid, was applied in parallel for comparison. Gene expression was induced by addition of IPTG to a final concentration of 1 mM. Cells were incubated overnight at 22 °C, harvested by centrifugation, and lysed in RIPA buffer (Thermo Fisher Scientific, Waltham, USA) with protease inhibitor cocktail (Roche) as described previously^53^. The production of EloRheb2 and EloNMT proteins was checked by immunoblotting using mouse anti-His (1:1,000, Invitrogen, Carlsbad, USA) and rabbit anti-HA (1:1,000, Sigma-Aldrich) antibodies, respectively. For detection, the HRP-labelled sheep anti-mouse (1:5,000, GE Healthcare Bio-Sciences, Pittsburgh, USA) and donkey anti-rabbit (1:5,000, GE Healthcare Bio-Sciences) secondary antibodies were used. The membranes were treated with ECL™ Western Blotting Analysis System (GE Healthcare Bio-Sciences).

The EloRheb2 was purified on the S-protein agarose (Merck Millipore). Its N-myristoylation was confirmed by mass spectrometry by a commercial service of the Proteomics Core Facility of the Central European Institute of Technology, CEITEC, Brno, Czech Republic using a standard protocol. Briefly, bands were dissected from a 1D polyacrylamide gel in the region corresponding to the expected size of the tagged EloRheb2 protein, destained, washed, and incubated with trypsin. Extracted tryptic peptides were analysed by liquid chromatography–tandem mass spectrometry (LC–MS/MS) using the RSLCnano system (Thermo Fisher Scientific) on-line coupled with Impact II Ultra-High Resolution Qq-Time-Of-Flight mass spectrometer (Bruker, Bremen, Germany). Pre-processing of the mass spectrometric data was carried out in DataAnalysis software version 4.2 SR1 (Bruker). Exported MS/MS spectra were analysed in Proteome Discoverer software v. 1.4 (Thermo Fisher Scientific) with in-house Mascot version 2.5.1 (Matrixscience, London, UK) search engine. Mascot MS/MS ion searches were done against an in-house database containing the predicted protein sequence of EloRheb2. Mass tolerance for peptides and MS/MS fragments were 25 ppm and 0.05 Da, respectively. Oxidation of methionine, carbamidomethylation (C), deamidation (N, Q), N-terminal acetylation, and N-myristoylation as optional modifications and two missed enzyme cleavages were set for all searches.

## Electronic supplementary material

Supplementary tables S1-S4

Supplementary figures S1-S7
