# Supplementary material for: Extensive molecular tinkering in the evolution of the membrane attachment mode of the Rheb GTPase
Source: Sci Rep. 2018 Mar 27;8:5239. doi: 10.1038/s41598-018-23575-0 (PMC5869587; doi:10.1038/s41598-018-23575-0)
Supplement: Supplementary file 2 — Supplementary figures S1-S7 [file 41598_2018_23575_MOESM2_ESM.pdf]

Extensive molecular tinkering in the evolution of the membrane attachment mode of the Rheb GTPase

Kristína Záhonová[1], Romana Petrželková[1], Matus Valach[2], Euki Yazaki[3], Denis V. Tikhonenkov[4], Anzhelika Butenko[1], Jan Janouškovec[5], Štěpánka Hrdá[6], Vladimír Klimeš[1], Gertraud Burger[2], Yuji Inagaki[7], Patrick J. Keeling[8], Vladimír Hampl[6], Pavel Flegontov[1], Vyacheslav Yurchenko[1], Marek Eliáš[1*]

[1]Department of Biology and Ecology & Institute of Environmental Technologies, Faculty of Science, University of Ostrava, Ostrava, Czech Republic

[2]Department of Biochemistry and Robert-Cedergren Centre for Bioinformatics and Genomics, Université de Montréal, Montreal, Canada

[3]Institute for Biological Sciences, University of Tsukuba, Tsukuba, Japan

[4]Laboratory of Microbiology, Papanin Institute for Biology of Inland Waters, Russian Academy of Sciences, Borok, Russia

[5]Department of Genetics, Evolution and Environment, University College London, London, United Kingdom

[6]Department of Parasitology, Faculty of Science, Charles University, Prague, Czech Republic

[7]Center for Computational Sciences, University of Tsukuba, Tsukuba, Japan

[8]Department of Botany, University of British Columbia, Vancouver, Canada

[*]Author for correspondence: Marek Eliáš, Department of Biology and Ecology, University of Ostrava, Chittussiho 10, 710 00 Ostrava, Czech Republic, tel.: +420-597092329, e-mail: marek.elias@osu.cz

Supplementary Figures S1 to S7

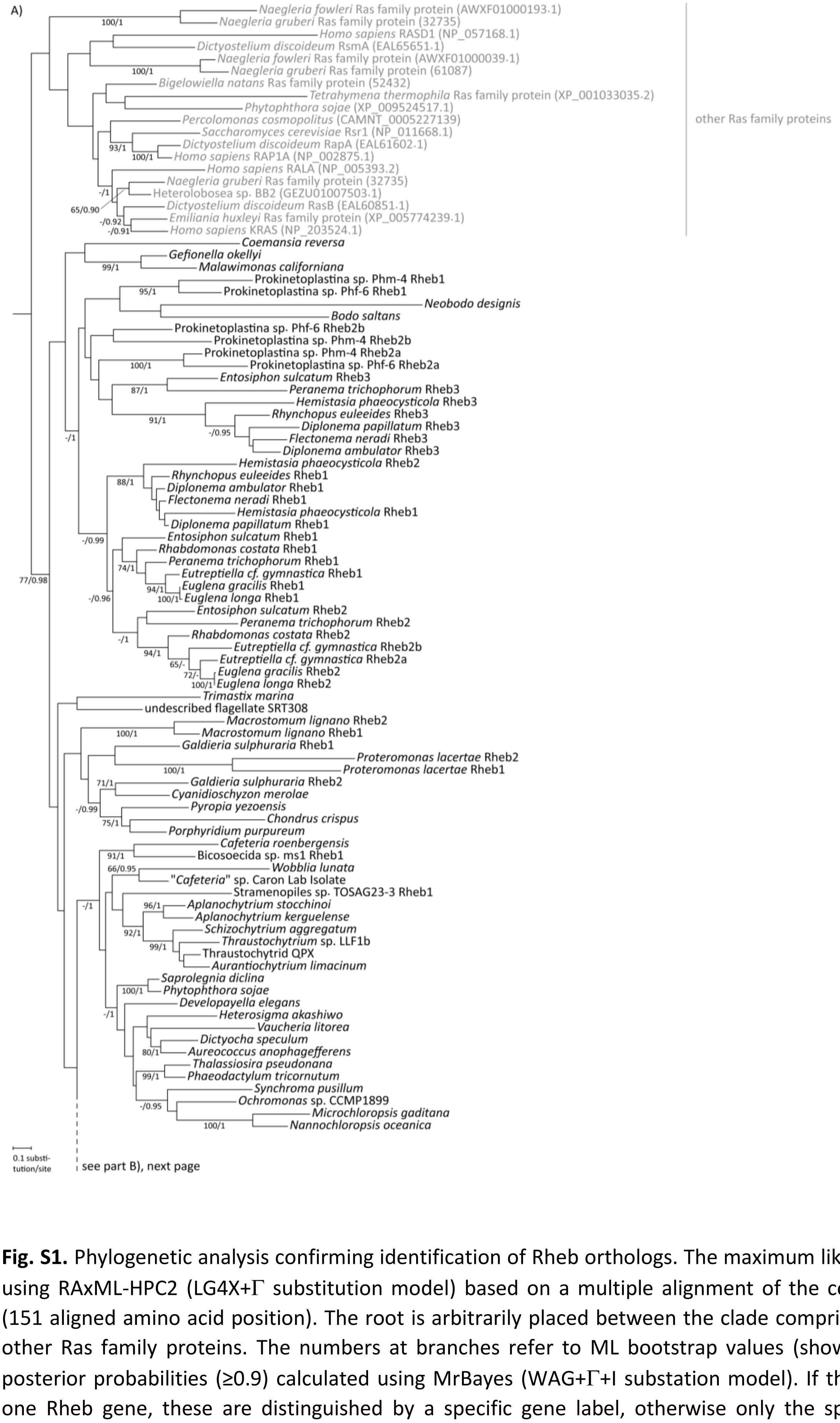

Fig. S1. Phylogenetic analysis confirming identification of Rheb orthologs. The maximum likelihood tree was inferred using RAxML-HPC2 (LG4X+Γ substitution model) based on a multiple alignment of the conserved GTPase domain (151 aligned amino acid position). The root is arbitrarily placed between the clade comprising bona fide Rhebs and other Ras family proteins. The numbers at branches refer to ML bootstrap values (shown only when ≥65%) and posterior probabilities (≥0.9) calculated using MrBayes (WAG+Γ+I substation model). If the species has more than one Rheb gene, these are distinguished by a specific gene label, otherwise only the species name is indicated. Sequence identifiers of the Rheb sequences included in the analysis is provided in table S1, those of other Ras family proteins are indicated in brackets (the respective databases are listed in table S1). Note the position of the *Naegleria gruberi* protein “61087” (among “other Ras family proteins”), which seems to have been misinterpreted as a Rheb ortholog by van Dam et al. (2011b).

B)

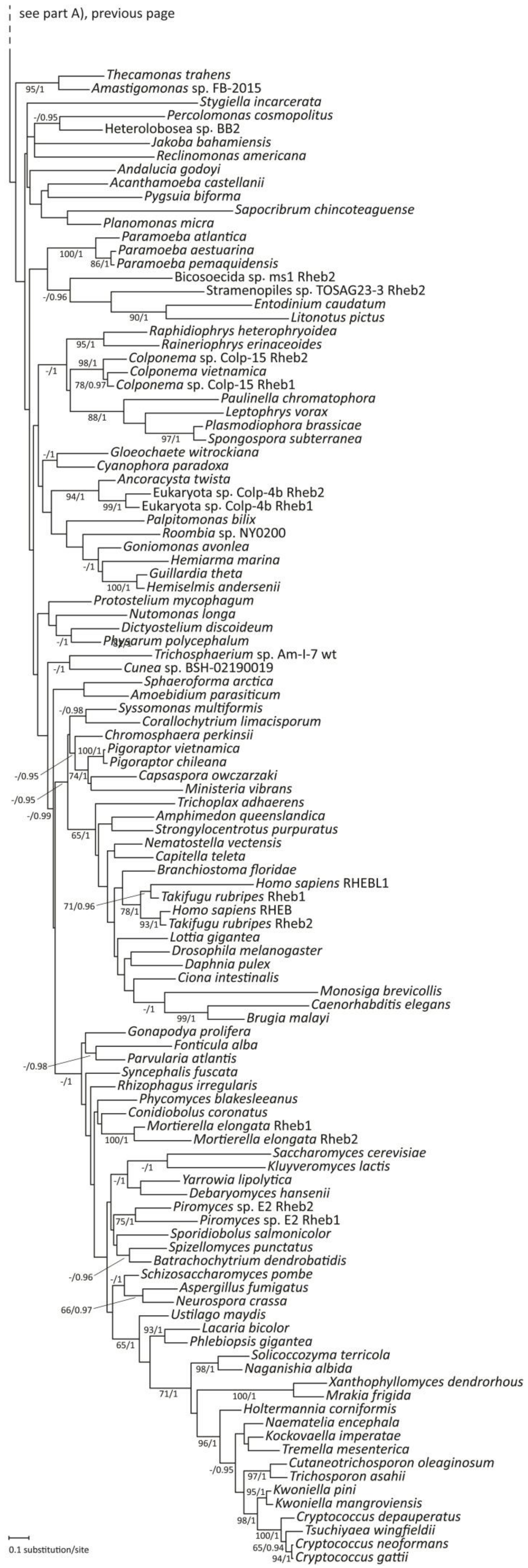
see part A), previous page
Thecamonas trahens
Amastigomonas sp. FB-2015
Stygiella incarcerata
Percolomonas cosmopolitus
Heterolobosea sp. BB2
Jakoba bahamiensis
Reclinomonas americana
Andalucia godoyi
Acanthamoeba castellanii
Pygsuia biforma
Sapocribrum chincoteaguense
Planomonas micra
Paramoeba atlantica
Paramoeba aestuarina
Paramoeba pemaquidensis
Bicosoecida sp. ms1 Rheb2
Stramenopiles sp. TOSAG23-3 Rheb2
Entodinium caudatum
Litonotus pictus
Raphidiophrys heterophryoidea
Raineriophrys erinaceoides
Colponema sp. Colp-15 Rheb2
Colponema vietnamica
Colponema sp. Colp-15 Rheb1
Paulinella chromatophora
Leptophrys vorax
Plasmodiophora brassicae
Spongospora subterranea
Gloeochaete witrockiana
Cyanophora paradoxa
Ancoracysta twista
Eukaryota sp. Colp-4b Rheb2
Eukaryota sp. Colp-4b Rheb1
Palpitomonas bilix
Roombia sp. NY0200
Goniomonas avonlea
Hemiarma marina
Guillardia theta
Hemiselmis andersenii
Protostelium mycophagum
Nutomonas longa
Dictyostelium discoideum
Physarum polycephalum
Trichosphaerium sp. Am-I-7 wt
Cunea sp. BSH-02190019
Sphaeroforma arctica
Amoebidium parasiticum
Syssomonas multiformis
Corallochytrium limacisporum
Chromosphaera perkinsii
Pigoraptor vietnamica
Pigoraptor chileana
Capsaspora owczarzaki
Ministeria vibrans
Trichoplax adhaerens
Amphimedon queenslandica
Strongylocentrotus purpuratus
Nematostella vectensis
Capitella teleta
Branchiostoma floridae
Homo sapiens RHEBL1
Takifugu rubripes Rheb1
Homo sapiens RHEB
Takifugu rubripes Rheb2
Lottia gigantea
Drosophila melanogaster
Daphnia pulex
Ciona intestinalis
Monosiga brevicollis
Caenorhabditis elegans
Brugia malayi
Gonapodya prolifera
Fonticula alba
Parvularia atlantis
Syncephalis fuscata
Rhizophagus irregularis
Phycomyces blakesleeanus
Conidiobolus coronatus
Mortierella elongata Rheb1
Mortierella elongata Rheb2
Saccharomyces cerevisiae
Kluyveromyces lactis
Yarrowia lipolytica
Debaryomyces hansenii
Piromyces sp. E2 Rheb2
Piromyces sp. E2 Rheb1
Sporidiobolus salmonicolor
Spizellomyces punctatus
Batrachochytrium dendrobatidis
Schizosaccharomyces pombe
Aspergillus fumigatus
Neurospora crassa
Ustilago maydis
Lacaria bicolor
Phlebiopsis gigantea
Solicoccozyma terricola
Naganishia albida
Xanthophyllomyces dendrorhous
Mrakia frigida
Holtermannia corniformis
Naematelia encephala
Kockovaella imperatae
Tremella mesenterica
Cutaneotrichosporon oleaginosum
Trichosporon asahii
Kwoniella pini
Kwoniella mangroviensis
Cryptococcus depauperatus
Tsuchiyaea wingfieldii
Cryptococcus neoformans
Cryptococcus gattii
0.1 substitution/site

```
                                                              <<<<<<<<<<<<<<<<<<<<<<<<<<<<<<<<<<<<<<<<<<<<<    PX domain    >>>>
Guillardia theta            M---------------ERSTRVQ---------SATITGTE-VREEGGKKFTVYKVELRSEIGPWVVWRRYRQFHELDAKIKERNPSFPGRLPQKKMGGNMK
Hemiselmis andersenii       M---------------ERTTRVQ---------SAMILGTE-NRDQGGKKFIVYKVELRSDIGPWIVYRRYRQFFELDTKLRERFPGFQGQLPKKKVSGNLA
Goniomonas avonlea          M---------------AAVDRPPPRSIG--SLRTRIVGSE-TRED-VSSFTVYQVQIRNEAGMWTVLRRYSQFYELVQKLRDRMPDRKWDFPKKKLTGNLE
Hemiarma marina             M-------------SMSRDSRRQPGSLSGGEFSARVGSWE-TRESGGKTFTVYKVKIQSELGSWTVARRYSQFDDLNKRLAISNPSAALPLPKKKLVGNQN
Roombia sp. NY0200          MAETAFGARSFGHSSHSQDDVFRSSS-SEMERKAEIVGSE-TRESPQKPFTVYTIQVTHGSSAWTIHRRYNQFFNLFNKLREQFPSIDFHFPPKKAFRNME
Palpitomonas bilix          M------------------RRRDPERLT----TLRITRTQLVKQEGGKGHIAYEILCAEGHQQWSTLKRYSELKELDGQIQRAFPSSSMPFPPKRLFGNMK
Homo sapiens RHEB           M----------------------------------------------------------------------------------------------------
Consensus/80%               M.................p...........ph.lh..p.s+pp..p.ahsYplbh.p....W.hh+RYppb.pL..pl....Ps..h.bP.K+h..Nb.

                            >>>>>>>>>>>>>>>>>>>>>>>>>>>>>>>>>>>                             <<<<<<<<<<<<<<<<<<<<<<<<<<<<<<<<<<<<<
Guillardia theta            PEFVEERKNFLQQYLKDLVADPNAAGSPEFRAFIDANRSCSGKLSDA-LGSGSGASSSTKQKLQRKIAVLGFMGVGKSAVTIQFTEGHFAEPYSPTIENTF
Hemiselmis andersenii       PEFVEERKRGLERYLEEVVSDANAVSSPDFRAFIDANRNCSAKLSEA-VAGNSVSGGGSRQRLQRKIAVLGFMGVGKSAVTIQFTEGHFAEPYSPTIENTF
Goniomonas avonlea          PAFVADRMKQLEAFLNEVVADHEVMRSPEFRAFIDANLDVSTKHSKL---SNSTEARSKRPMLSRKLAVLGFMGVGKSAVTIQFAEGHFPEIYSPTIENTF
Hemiarma marina             KDFIEQRRVALDKYVREICDDPRLANSQEFRSFIDTNLEAHTRVSKAGLSVSAQDGSSNGSGVPRKICVLGYMGVGKSCLTIQFTEGHFVDTYSPTIENTF
Roombia sp. NY0200          SDVIQQRKQHLQQFLDLVVAEQMIFESSGVVYFFDRSVDVSYNNGDD------RSSSKKNALKVRRLAVVGFMSVGKTAVSTQFVDEQFSEQYVPTIENTH
Palpitomonas bilix          EAFVKERSEALQAYFDGIIADEKMAGTEFVRSFFRRAEARPQGQGGG-SATATPTVDGSKLKRNRKVALLGFASVGKSAIAVQFAEEHFAEPYNPTIENTF
Homo sapiens  RHEB          -----------------------------------------------------------PQSKSRKIAILGYRSVGKSSLTIQFVEGQFVDSYDPTIENTF
Consensus/80%               ..hl.pR...Lp.ahp.lhs-..hh.*..h..Fbc.s.......t..........s..p...b.RKlAlLGabtVGKStl*lQFsE.pFs-.YsPTIENTF

                            <<<<<<<<<<<<<<<<<<<<<<<<<<<<<<<<<<<<<<<    Rheb GTPase domain    >>>>>>>>>>>>>>>>>>>>>>>>>>>>>>>>>>>>
Guillardia theta            QKVIRHKGIEYQTDILDTAGQDEYQIFHTRYAMGIHGYILMYSIVSRRSFDMVSTIRDKLLNAVGTENIPHVLVGNKSDLVHEREVATEEGQRLADEWGVP
Hemiselmis andersenii       QKVIRHKGTEYQTDILDTAGQDEYQIFHTRYAMGIHGYILMYSVISRRSFDMVSTIRDKLLNAVGTEDIPHVLVGNKSDLVHEREVTKEEGQRLADDWGVP
Goniomonas avonlea          QKLIKYRGVEYATDILDTAGQDEYQIFHTRYAMGIHGYVLVYAVSVRRSFDMIRTIHEKLVNAMGTDQIPRILVGSKRDLTHQREVTPEEGQRLADEWGCP
Hemiarma marina             SKQLKYRGVDYSVEILDTAGQDEYHIFHTRYAMGVHGYVLMYTVNLRRSFEMVETIHDKLVNAIGTSQVPRVLVGAKCDLVHERQVGSDEGYALAQQWGCP
Roombia sp. NY0200          HKVVTHRGEEFVLDIVDTAGQDDSQILHQRYTIGVHGYALVFSVASRRSFEMIEVIHEKLVNALGHDRFPKVLVGNKVDLTVQREVSTEDAQRLADSWGCL
Palpitomonas bilix          HKTIKHKGIEYAFDVVDTAGQEEMSQFPSRHCIGVHAYVLVYSVSSRRSFDMIPVINTKLLNATGNDQAVRILVGNKTDLVLDRKVTTEEGEAMAAKYNMK
Homo sapiens RHEB           TKLITVNGQEYHLQLVDTAGQDEYSIFPQTYSIDINGYILVYSVTSIKSFEVIKVIHGKLLDMVGKVQIPIMLVGNKKDLHMERVISYEEGKALAESWNAA
Consensus/80%               pKhlpa+G.EY.h-llDTAGQDEbpIF.pRYsbGlHGYlLhY*VssRRSF-MlpsI+pKLlNAhGppphP+lLVGsKpDLsbpRpVs.EEGp.LAppWss.

                            >>>>>>>>>>>>>>>>>>>>>               CaaX
Guillardia theta            FIESSAKFNTNIEEIFRKLILQCEA-GQEGSDGEKKCLIM
Hemiselmis andersenii       FLESSAKFNTNVEEVFKRLIAQTEA-GQDNGKGDKNCTVC
Goniomonas avonlea          FLECSAKYNENIGDVFNKLIAEIERFNNPNPEQQETCCIA
Hemiarma marina             FFECSAKLNEKIDDIFLSLLAEIDK-QREPEQQHESCTMQ
Roombia sp. NY0200          LLECSAKDGDNISDVFSKLLAEIEK--AAAPEEKDTCIIF
Palpitomonas bilix          FLECSAKYNEHIWDIFKSILAELDK-TENPDQSDAGCSIM
Homo sapiens RHEB           FLESSAKENQTAVDVFRRIILEAEKMDGAASQGKSSCSVM
Consensus/80%               FlEsSAKbNppl.-lFppllhph-.....ssp.cpsC.lh
```

Fig. S2. Annotated multiple alignment of PX-Rheb protein sequences. Sequence accession numbers are provided in table S1. The alignment was prepared using MAFFT version 7 and processed for visualization using Chroma v1.0. The *Homo sapiens* RHEB sequence represents the conventional Rheb form lacking the N-terminal PX domain.

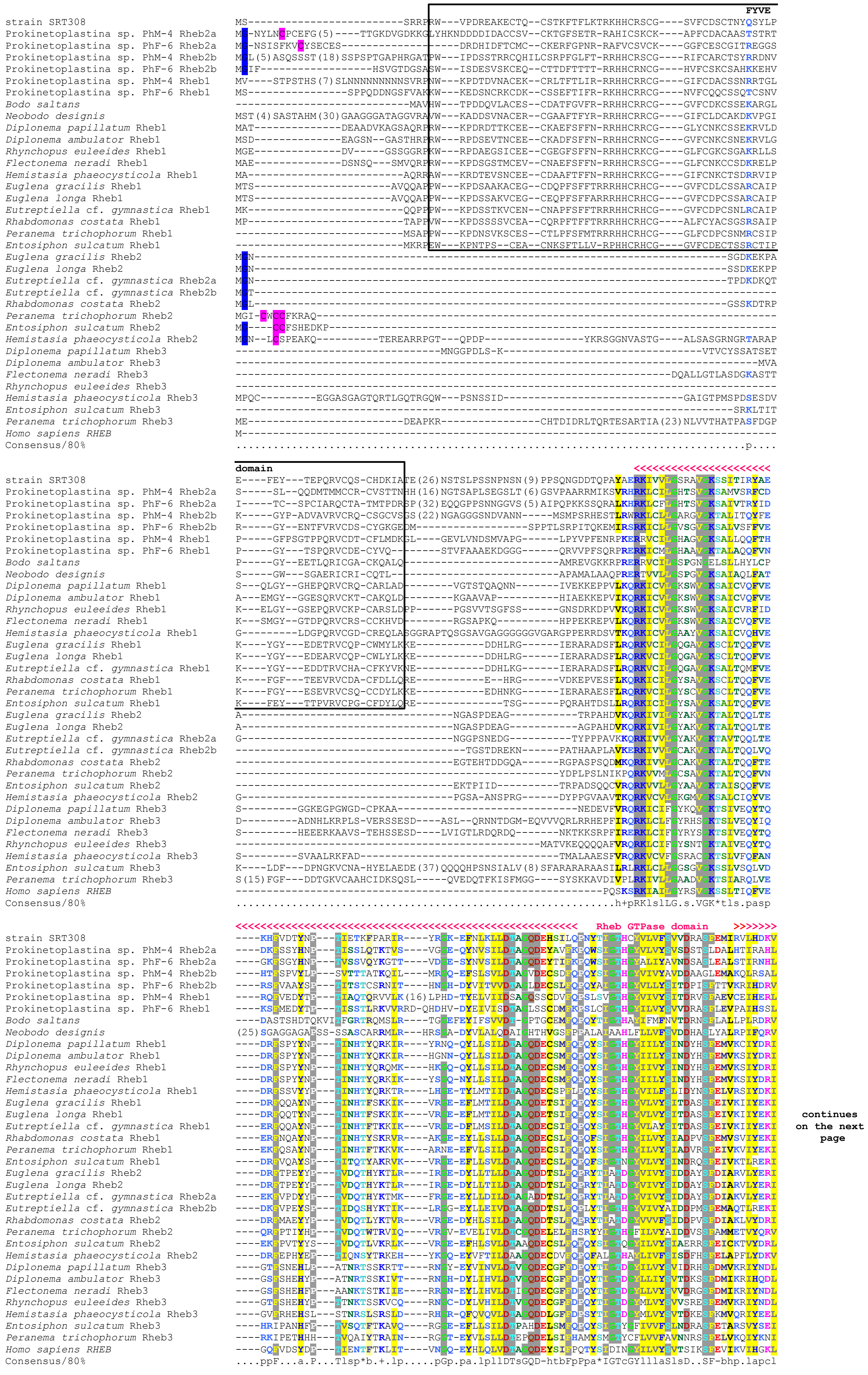
FYVE
domain
Rheb GTPase domain
continues on the next page

```
                                          >>>>>>>>>>>>>>>>>>>>>>>>>>>>>>>>>>>>>>>>>>>>>>>>>>>>>>>>>>>>>>>>>>>>>>>>>>>
strain SRT308                             LNACA(35)SK-VVIILVGNM----CDLER-----NRCVSNEEGEALARELGCSFIECSAKTDENIHKIFETAVHNYELRFNQ---E
Prokinetoplastina sp. PhM-4 Rheb2a        LECHA----PD-VPLVLCANK----CDTSE----MDRQVLSQDGLELAQRWRCDFVECSAKTGYNVPTVFEKLLTRIAQS-------
Prokinetoplastina sp. PhF-6 Rheb2a        LECHA----PD-VPIVLVANK----VDLPD----GEIQISEAQGREMAAKWKTSFVQCSAKTGEGVNEVFEALLRRIAHN-------
Prokinetoplastina sp. PhM-4 Rheb2b        LECYA----SG-VTMVLVGTK----ADVPA----SQRVVSSEEGHALAEMWCCPYVECSAATGSCVERMFAILLESIQLQENV---R
Prokinetoplastina sp. PhF-6 Rheb2b        LECHP----PG-IPIVLAGTK----ADARN----SERQVSEGEGKALSAVWGCPHVECSARTGINVDKVFLSLLEKIQKDD------
Prokinetoplastina sp. PhM-4 Rheb1         REC-H----PN-APVLVVGNK----SDLER---STERAITAAEGAALAAELSCGFLECTATKANVATAVFYTILDMIVAAEAV---V
Prokinetoplastina sp. PhF-6 Rheb1         AQC-H----ND-VPIVLVGNK----IDLVE--KAAERQVTSEEGAELAARLGCSFIECSATRQKAVQNVFSTILDLIVASEKD----
Bodo saltans                              LECGG----ME-KAMILVGNK(10)TNGPQ----LDRQVTVDEVSSLGHRWGVPHLELSSSKQRQVDELKNTLLRLVAERDHL---S
Neobodo designis                          LDCGC----HF-LPIIVVGTT-------RG----ESRVVPSEDCAAVAAEFGAKYVEITLTPGD-VAGLFERALDQVAKE-------
Diplonema papillatum Rheb1                QDCNI----LEGVVLVLVGNK----NDLPE----GERQVSYDEAQQLAASWGFPFVECSAKEAESVEGVFNLVLEEILKAEGD---A
Diplonema ambulator Rheb1                  QDCNI----LDGVVLVLVGNK----SDLPA----SERQVSYEEGQQLAQSWGFPFMECSAKNAENIERVFNLVLEEILNAEGE---G
Rhynchopus euleeides Rheb1                QDCNI----LDGVVLILVGNK----SDLPS----SERQVSHEEGQQLAGSWGVPFMECSAKQSDTVEGVFNLMLEEILKAEGD---A
Flectonema neradi Rheb1                   QDCNI----RDGVVLVLVGNK----NDLPA-----ERQVSFEEGQQLAASWGFPFMECSAKKAENIEAVFNLVLEEILRAEGD---S
Hemistasia phaeocysticola Rheb1           QDCCN----IDGVPLVLVGNK----MDLRE-----DRKVHEAEAQALAAEWGVPYLECSAKDARSVELVFNTVLTEIVRLEAP---Q
Euglena gracilis Rheb1                    HSY-A----VD-LVLVLVGNK----TDLDH-----QRQVSHEEALKLAQEWRCPLIECSAKKKENISKVFYTIMEEILTQEDG---A
Euglena longa Rheb1                       HSY-A----VD-LVLVLVGNK----TDLDH-----QRQVSREEALKLAQEWRCPLIECSAKKKENISKVFYTIMEEILTQEDG---A
Eutreptiella cf. gymnastica Rheb1         HSY-A----VD-VVLVLVGNK----TDLEK-----DRQVTYDEAETLAREWNCPFIECSAKRKENIAKVFYTVMEAILTQEDG---V
Rhabdomonas costata Rheb1                 HSF-A----VD-VVLVLVGNK----CDLER-----DRQVSFEQGQALARGWKCPFIECSAKRKDNVAKVFLTILEEILTQEDG---G
Peranema trichophorum Rheb1               YSY-A----VD-VVLLLVGNK----SDLDK-----ERQVSFEEGSALAREWKCPFLECSAKKKDNVVRVFYGILEEILKAEDP---T
Entosiphon sulcatum Rheb1                 FSH-A----VD-VVLLLVGNK----SDLDK-----DRQVSVEAGKALAKEWDSPFMECTATKKEQVATVFQTVMEAILAHEEG---G
Euglena gracilis Rheb2                    HEY-V----VD-GVVVLLGNK----TDLEK-----DRQVSYQQAQALAEEWKCPFLECSAKRKENIQRVFTTIMEEILKREDT---P
Euglena longa Rheb2                       HEY-V----VD-GVVVLLGNK----TDLEK-----ERQVSYQQAQALAEEWKCPFLECSAKRKENIQRVFTTIMEEILKREDT---P
Eutreptiella cf. gymnastica Rheb2a        HEY-V----VE-GVVVLVGNK----VDLDA------RQVSHAQGQALADEWKCPFLECSAKRKENIQKVFTIMLEEILKREDA---P
Eutreptiella cf. gymnastica Rheb2b        QDSGV----VE-AVVVLVANK----TDLDA------RQVSHAEAQALAESWSCPFLECSATRKESVQRVFTTMLEQILQHEDA---P
Rhabdomonas costata Rheb2                 HEF-V----VD-AVIVLVGNK----TDLDR-----ERQITTEQGQALAQSWRCTYIECSAKKKENVNRIFQTILEQILSREEG---G
Peranema trichophorum Rheb2               CDN-V----LY-PTIVLVGNK----SDRQS-----HRKVTREQGQALANRWGCPFIESCAQQNESANVVFGAILDIILEREDS---P
Entosiphon sulcatum Rheb2                 HCE-V----VD-AAVVLVGNK----IDLEK-----QRQVPTEEAQALAQSWGCPFVEVSAKKKDSVNRIFTMLVDEILTREEG---S
Hemistasia phaeocysticola Rheb2           RDC-N----LDDVAVVLVGNK----SDLAG-----DREVPVEEAMRLAQSWGCPYVECSAKDSEQVRDVFVAVLGEILKREL-----
Diplonema papillatum Rheb3                WAY-T----PL-VPFVLVANK----SDLKA-----QVSVSGEEGWNLAKKWGCPYEEATSLSGERINEIFDKLLGRIDVSYDD---T
Diplonema ambulator Rheb3                  WNY-T----PL-VPFVLVANK----VDLVD-----HREVGEEEGRALAQKWACPYTEVTSRSNKEVTQLFSFLLSKIDTAYDD---T
Flectonema neradi Rheb3                   WAY-T----PL-VPFVLVANK----ADLTS-----DREVSEEEGLQLARRWNCDYAEVTSKSGYDIECVFSKLLLKIDTMYDD---T
Rhynchopus euleeides Rheb3                WAY-T----PL-VPFVLVATK----VDLTH-----ERVVTEQDGRALAAQWGCDYFEVTSRTGKSAANMIAQLLEKIDAMYDD---S
Hemistasia phaeocysticola Rheb3           WAY-T----PL-VPFILVANK----VDLAT-----QREVTAREGQRLARQWGCHYFESTAKDADCVHAAFEALLAQIDGSYEQ(6)T
Entosiphon sulcatum Rheb3                 MAN-T----VG-IPILLVGNK----SDLCR(10)GAGAVGTEEGMRLASEWDAQYMECTATDHFSAHAVFMRMLDMIEGPLSL(6)S
Peranema trichophorum Rheb3               QEN-T----VC-VPIVLVGNK----SDLP------NREVSQEEADELANSWFCKYVETTALNYSNIRKLIDLAMDEIEGPLET---K
Homo sapiens RHEB                         LDM-V--GKVQ-IPIMLVGNK----KDLHM-----ERVISYEEGKALAESWNAAFLESSAKENQTAVDVFRRIILEAEKMDGA---A
Consensus/80%                             bph.s.......ssllLVtNK....sDL.......pRpV*.pptb.LA.pW.ssahEs*App.psl..lF..llppI...b......

strain SRT308                             AYH--------QETPPT-CLLL--------------
Prokinetoplastina sp. PhM-4 Rheb2a        ------------------------------------
Prokinetoplastina sp. PhF-6 Rheb2a        ------------------------------------
Prokinetoplastina sp. PhM-4 Rheb2b        E-----------------------------------
Prokinetoplastina sp. PhF-6 Rheb2b        -----------------------------------V
Prokinetoplastina sp. PhM-4 Rheb1         C-----------------------------------
Prokinetoplastina sp. PhF-6 Rheb1         -----------------------------------D
Bodo saltans                              G-----------------------------------
Neobodo designis                          ------------------------------------
Diplonema papillatum Rheb1                ------------------------------------
Diplonema ambulator Rheb1                 S-----------------------------------
Rhynchopus euleeides Rheb1                ------------------------------------
Flectonema neradi Rheb1                   ------------------------------------
Hemistasia phaeocysticola Rheb1           ------------------------------------
Euglena gracilis Rheb1                    PKQPAK---------------PFWRIA---------
Euglena longa Rheb1                       PKQPIK---------------PFWRIG--------Q
Eutreptiella cf. gymnastica Rheb1         PKQTSK---------------MFWRKA-------DR
Rhabdomonas costata Rheb1                 PRPSRW---------------PSWLRF---------
Peranema trichophorum Rheb1               PKKEGKD-----KAKPK-GKGLF-------------
Entosiphon sulcatum Rheb1                 QRRLPP--------TPK-K-----------------
Euglena gracilis Rheb2                    ATAGDRP-----AAKPF-WKGMFQS----------G
Euglena longa Rheb2                       ATAGDRP-----PIKPF-WMGMFQS----------S
Eutreptiella cf. gymnastica Rheb2a        CCAGDRP-----QSRPF-WTGMFHK----------S
Eutreptiella cf. gymnastica Rheb2b        FSSGQSA----HECSSI-WKAIF-------------
Rhabdomonas costata Rheb2                 GAGAKQSKGLR---------GFFGK----------P
Peranema trichophorum Rheb2               T-----------PTSGT-SSSFLHKIM-------GT
Entosiphon sulcatum Rheb2                 A-----------RVRGS-W---FNKTP--------R
Hemistasia phaeocysticola Rheb2           ------------------------------------
Diplonema papillatum Rheb3                PEFAK--(4)FGTG----------------------
Diplonema ambulator Rheb3                 ETFIR--(4)NTAPRPS-CALQ--------------
Flectonema neradi Rheb3                   ETFVK--(2)SQANQPS-CSVM--------------
Rhynchopus euleeides Rheb3                DTFFK--(3)--RQQQS-CSVM--------------
Hemistasia phaeocysticola Rheb3           ETFAPTE(10)QRSKDG-CSVM--------------
Entosiphon sulcatum Rheb3                 TTTTRNN(71)REEEPA-ASVVAAEKRSAAKGRTGE
Peranema trichophorum Rheb3               DPGVRRC(14)AESEGYRCVIQ--------------
Homo sapiens RHEB                         S-----------QGKSS-CSVM--------------
Consensus/80%                             ....................................
```

Fig. S3. Annotated multiple alignment of Rheb protein sequences from Euglenozoa. Sequence accession numbers are provided in table S1. The alignment was prepared using MAFFT v7, manually adjusted, and processed for visualization using Chroma v1.0 (numbers in brackets indicate the number of amino acid residues omitted from the display for simplicity). The *Homo sapiens* RHEB sequence represents the conventional Rheb form. Positions predicted to be N-myristoylated (see table S3) are highlighted with a blue background, positions predicted to be S-palmitoylated (see table S4) are highlighted with a pink background, the CaaX box is highlighted with a red background. Note that the sequence of the C-terminus of the Rheb3 protein from *Entosiphon sulcatum* has not been defined (the available transcript sequence is either 3'-truncated or has a retained intron), so it is unclear whether this protein has the CaaX box (as its orthologs) or lacks it. The region corresponding to the FYVE domain is boxed. Note that Pfam search (http://pfam.xfam.org/) failed to recognize the FYVE domain in the Rheb1 proteins from the two Prokinetoplastina species, but the presence of the domain (albeit possibly divergent) in these two proteins is supported by sequence conservation in the respective region and searches against the NCBI Conserved Domains database (https://www.ncbi.nlm.nih.gov/Structure/cdd/wrpsb.cgi).

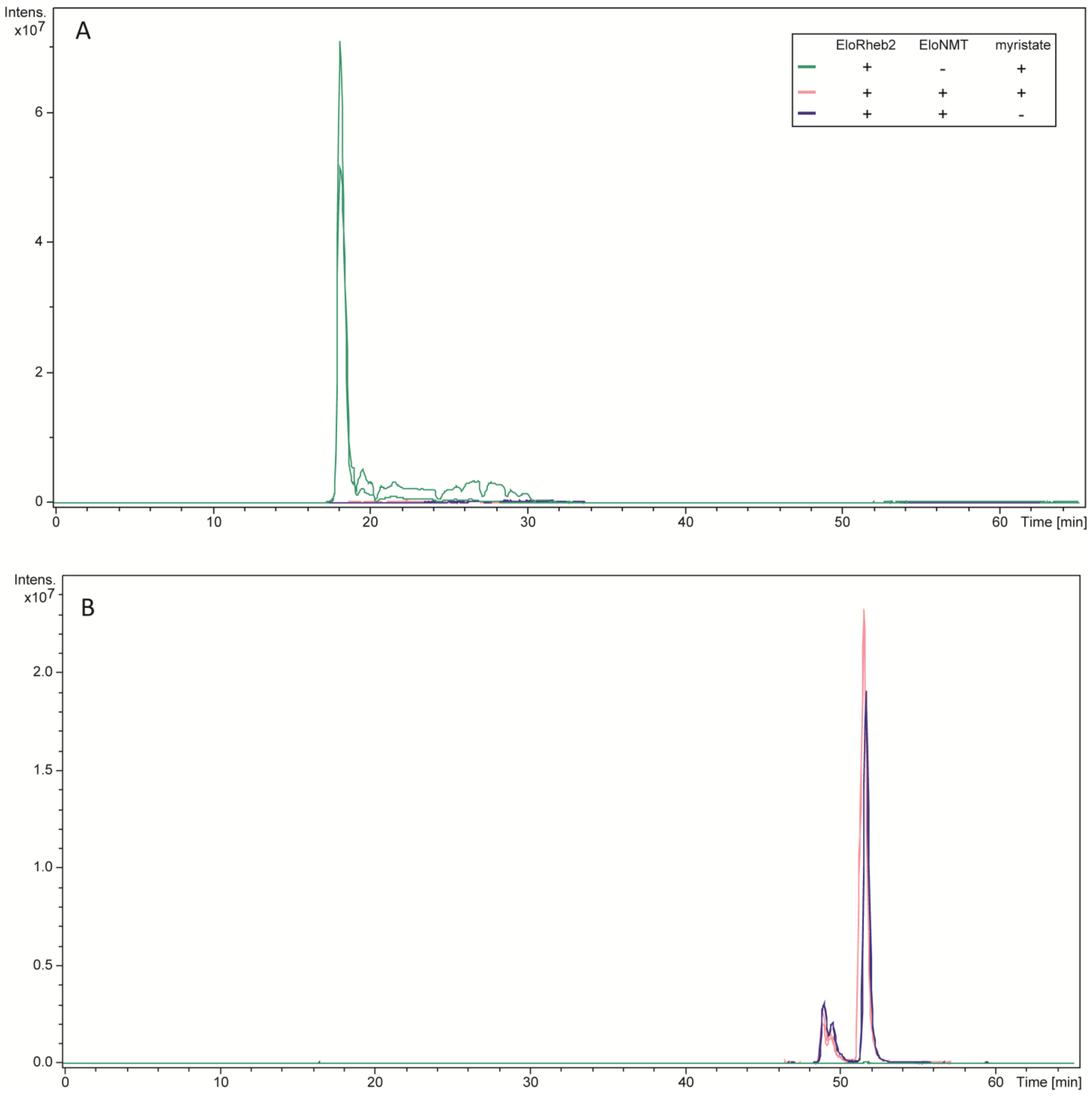

Fig. S4. N-terminal myristoylation of the *Euglena longa* Rheb2 protein detected by mass spectrometry. EloRheb2 protein was affinity purified on S-protein agarose, dissected from a gel after 12% SDS-PAGE electrophoresis, cleaved with trypsin, and the peptides were analyzed by mass spectrometry. The plots shows the signal for two different variants of the N-terminal tryptic peptide (GNSSDKEKPPANGASPDEAGAGPAHDVK) of the Rheb2 protein: A) an unmodified form (2702.25 Da, a lower LC-MS retention time); B) a myristoylated form (2912.45 Da, a higher LC-MS retention time). Both peptide variants were monitored in three different samples (each sample processed twice, the results shown as two lines of the same colour): green, samples from *E. coli* expressing Rheb2, but not N-myristoyl transferase (NMT), supplemented with myristate; pink and blue, samples from *E. coli* expressing Rheb2 and NMT in the presence or absence of external myristate, respectively. Note the lack of a green signal for the myristoylated peptide (consistent with the lack of myristoylation in the absence of NMT). The lack of a pink and a blue signal for the non-myristolated peptide indicates a high efficiency of NMT-dependent N-myristoylation in the heterologous system of *E. coli* cells, even in the absence of the external myristate.

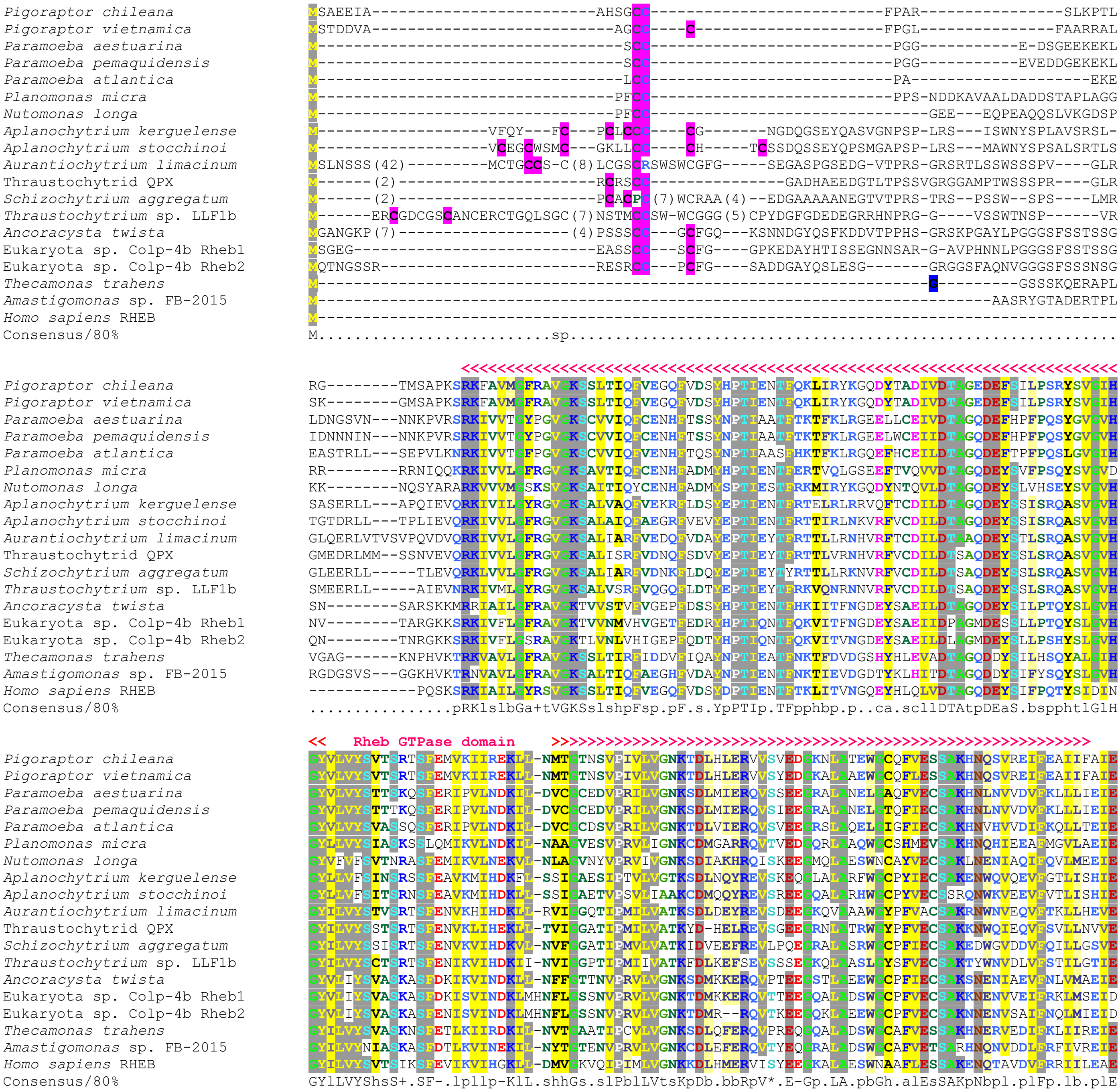

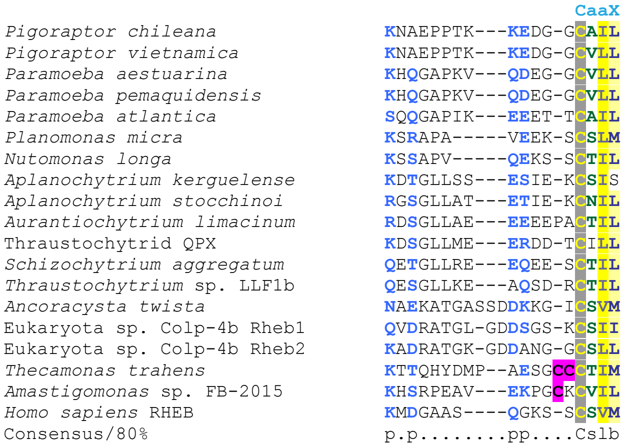

Fig. S5. Annotated multiple alignment of Rheb protein sequences with putative S-palmitoylation (excluding dually acylated Rhebs from Euglenozoa). Sequence accession numbers are provided in table S1. The alignment was prepared using MAFFT v7, manually adjusted, and processed for visualization using Chroma v1.0 (numbers in brackets indicate the number of amino acid residues omitted from the display for simplicity). The *Homo sapiens* RHEB sequence represents the conventional Rheb form lacking an acylated N-terminal extension or palmitoylated cysteines adjacent to the C-terminal CaaX box. Positions predicted to be S-palmitoylated (see table S4) are highlighted with a pink background. Note also the putatively myristoylated glycine residue (highlighted with a blue background; see also table S3) at the N-terminus of the Rheb sequence from *Thecamonas trahens*.

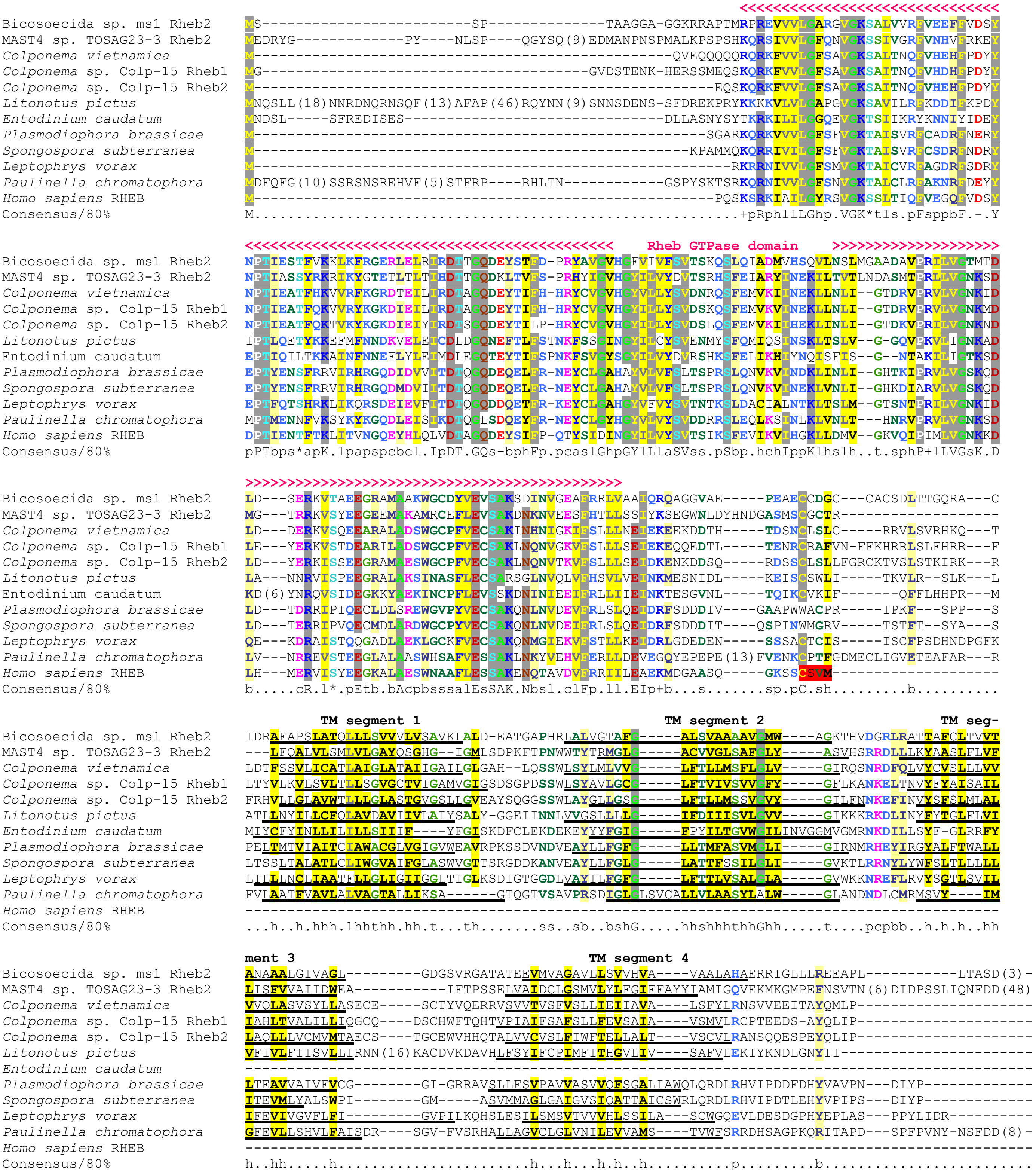

Fig. S6. Annotated multiple alignment of Rheb-TM protein sequences from the SAR clade. Sequence accession numbers are provided in table S1. The *Homo sapiens* RHEB sequence represents the conventional Rheb form with the C-terminal CaaX box (highlighted in red). The alignment was prepared using MAFFT version 7, manually adjusted, and processed for visualization using Chroma v1.0 (numbers in brackets indicate the number of amino acid residues omitted from the display for simplicity). Underlined regions correspond to trans-membrane (TM) segments as predicted using the TMHMM Server 2.0 except for the last TM segment in the Rheb sequence from *Leptophrys vorax*, which was not recognized by TMHMM but was predicted by the alternative tool TMpred. The Rheb sequence from *Entodinium caudatum* lacking the last two transmembrane segments may not be complete. Note that some of the Rheb-TM sequences still exhibit a cysteine residue at the position corresponding to the cysteine residue of the CaaX box of conventional Rheb (suggesting that Rheb-TM emerged via direct accretion of the C-terminal region with the transmembrane segments to the original C-terminus).

```
                                                                                       W-rich
                                                                                       motif
Cryptococcus neoformans          116 VPLVIVGQKTDLR-DERSVTKEEGEALAKKLRAGFIESSAKDNANVSGAFEVLLEEMQKVYN---PQ-PEKKKS-SWWP-W-----
Cryptococcus gattii              116 VPLVIVGQKTDLR-DERSVTKEEGEALAKKLNAGFIESSAKDNANVSGAFEALLEEMQKVYN---PQ-PEKKKS-GWWP-W-----
Cryptococcus depauperatus        115 MPLVIVGQKTDLR-DERSVTREEGEALAKKLKAGFVESSAKDNNNVSAAFETLLEEMQKVYN---PQ-PEKKKS-GWWP-F-----
Tsuchiyaea wingfieldii           117 VPLVIVGQKTDLR-DQRSITKDEGEALAKKLHAGYVESSAKDNNNVGVAFEMLLNEMQRVYN---PE-PEKKKS-GWWP-F-----
Kwoniella pini                   260 VPCVIVGQKVDLQ-SERAITTAEGEALAKKLNAGFIESSAKDNKNVSKAFDVLLAEMQKEYN---PA-PEKKKS-SWWG-WGAK--
Kwoniella mangroviensis          274 VPCVVVGQKLDLQ-SERAVTTAEGEALAKKLNAGFIESSAKDNKNVSRAFDVLLAEMQKEYN---PA-PEKKKS-SWWS-WGAK--
Kockovaella imperatae            115 VPCVVVGQKVDLSGSERRVTFAEGEALAKKLHASFIESSAKDNKNVGKAFEVLLREMQKEYN---PA-PEKKKS-SWWG-WGK---
Naematelia encephala             115 VPCVIVGQKSDLSAADRRVSVAEGEALAKKLGASFVESSARDNKNVGKAFEILLQVMQKEYN---PK-PEKKKS-SWWP-FSS---
Tremella mesenterica             113 VPCVIVGQKCDLDSSERRVTTIEGQELAKKLKAGFVECSAKDNKNVGKAFEALMNEMRMAYN---PP-PEKKKS-SWWI-WGR---
Trichosporon asahii              271 VPCVIVGQKCDLK-DERRVTAAEGQALAKKLGAAFVESSAKDNKNVEGAFDALLAEMQREYN---PAPPAPAKS-KWWP-W-----
Cutaneotrichosporon oleaginosum  341 VPCVIVGQKVDLD-KERRVSKAEGEALAKKLGAAFLESSAKDNKNVDRAFEALLGEMQREYN---PA-PAPAKK-GWWP-WS----
Holtermannia corniformis         217 VPCVIVGQKSDLS-EARQVEADEGEALAKKLKAVFIETSAKDNKNVGKVFDLLLGEMQKVYN---PA-PEKKKS-SWFS-WGSK--
Solicoccozyma terricola          122 VCCVIVGQKSDLG-ENRQVSLEEGAGLAKKLGAAFIETSAKDNKHINDVFNLLMIEMRRVYN---PP-EEKKKV-SWWSSWVGGGK
Naganishia albida                147 VCCVVVGQKCDLN-GVRQVKFEEGQALAKKLHANFIETSAKDNKNITDVFDLLMVEMRKAFN---PP-PEKKKQ-GWFASWFGGA-
Xanthophyllomyces dendrorhous    114 APCVIVGNKSDLN-GNRAVPTAEAKAFAQRIKAPFVETSAVKNDKIDEPVQSLLAEIWKSEG(6)AA-PAKATGGSWFGSWFGGKK
Mrakia frigida                   117 VPCVVVGNKSDLD-AARVVSVAEGKAMAAKIKAPFVETSAVKNANIHEPVNLLLAEIYKSEG(4)AA-PAASGGGSWLGGWFGKK-
Homo sapiens RHEB                112 IPIMLVGNKKDLH-MERVISYEEGKALAESWNAAFLESSAKENQTAVDVFRRIILEAEKMDG---AA-SQGKSSCSVM--------
Consensus/80%                        VPhVlVGQKsDLp..pR.V*..EGpALAKKL.AsFlE*SAKDNpNls.sFchLl.EMp+.aN...P..P..pKt.tWas.a.....
```

Fig. S7. Annotated multiple alignment of the C-terminal region of Rheb protein sequences from Tremellomycetes. The *Homo sapiens* RHEB sequence represents the conventional Rheb form with the CaaX box at the C-terminus (highlighted in red). The numbers on the left indicate the position of the first amino acid residue shown in the original full-length sequence (for accession numbers of the sequences see table S1). The alignment was prepared using MAFFT version 7, manually adjusted, and processed for visualization using Chroma v1.0 (numbers in brackets indicate the number of amino acid residues omitted from the display for simplicity). Note the replacement of the CaaX box by a tryptophan-rich motif in all tremellomycete sequences.
